# Brain wiring economics, network organisation and population-level genomics

**DOI:** 10.1162/IMAG.a.31

**Published:** 2025-06-04

**Authors:** Alicja Monaghan, Danyal Akarca, Duncan E. Astle

**Affiliations:** MRC Cognition and Brain Sciences Unit, University of Cambridge, Cambridge; The Alan Turing Institute, London, United Kingdom; Department of Electrical and Electronic Engineering, Imperial College London, London, United Kingdom; Department of Psychiatry, University of Cambridge, Cambridge, United Kingdom

**Keywords:** structural connectivity, generative modelling, polygenic scores, general intelligence, the ABCD study, graph theory

## Abstract

What role do our genes play in shaping the structural organisation of the living human brain? Across a sample of 2,153 children (9–11 years old), we address this question, focusing on common genetic variants associated with cognitive ability and diffusion-based structural neuroimaging. Using polygenic scores, we test how variability in the genetic signal associated with cognitive ability is linked to simulated structural network properties, such as network efficiency. We fit a computational model to each connectome that simulates the emergence of high-level network properties. Central to the model is an economic trade-off between the “cost” of forming a given connection (a distance penalty) and the topological “value” that connection brings to the network. To simulate the network properties of those with the highest genetic propensity for cognitive ability, we had to use a significantly weaker*wiring cost*penalty. This softer distance penalty produces more stochastic, diverse, and efficient simulated networks. Further, those with a high genetic propensity for cognitive ability exhibited a more randomised simulated topology. Finally, we took a different approach to exploring the relationships between genes and model parameters by linking the*distribution*of those parameters with post-mortem gene expression data, with a comparative pathway enrichment analysis. Across the sample, overlapping biological and cellular pathways between polygenic scores and each child’s optimal cost-value trade-off emerged. Together, the generative wiring distance term, which varied maximally across participants but minimally across the cortex, was enriched for more ontologies than the wiring value term, which varied maximally across the cortex. However, the overlap in enriched ontologies between polygenic scores and the wiring*value*term was greater than that of polygenic scores and the wiring*distance*term. This application of computational modelling demonstrates that the underlying economic trade-offs needed to simulate the higher-order topological properties of networks vary according to genetic propensity for cognitive ability.

## 1 Introduction

During development, the human brain organises itself across multiple spatial and temporal scales, supporting an increasingly elaborate cognitive and behavioural repertoire. The resulting organisation of the brain is variable across individuals, and differences in that organisation are associated with several broad domains of cognition, including executive functioning, memory, language, and fluid reasoning (Jung & Haier, 2007;Siugzdaite et al., 2020). But what factors shape brain organisation, and its variability, in the first place?

One approach to studying the structural organisation of the brain is to represent it as a connectome, with each brain region represented as a node connected by edges (Bullmore & Sporns, 2009). Graph theory, established byEuler (1741), describes these networks mathematically, capturing their organisational principles (seeBullmore & Sporns, 2009). The human brain has several organisational hallmarks consistent across scales, such as being “small world” (Watts & Strogatz, 1998), balancing network efficiency with wiring costs (Kaiser & Hilgetag, 2004).

Graph theory*describes*organisational features of networks, but can we*simulate*the formation of those features using some simple mathematical rules? Generative network models (GNMs) simulate the formation of complex networks from a sparse “seed” network, upon which connections are added probabilistically. The model’s first instantiation added connections in order to minimise wiring costs (Kaiser & Hilgetag, 2004), with subsequent extensions adding a second constraint (Vértes et al., 2012;Betzel et al., 2016), where nodes with specific properties wire together preferentially, to maximise efficiency. Put simply, these models compress complex topology to a relatively simple economic trade-off, which unfolds iteratively. Of course, the model is not designed to tell us*how*specific new connections are formed but is nonetheless a powerful way of testing how that economic trade-off—between connection “cost” and “value”—might operate to create differences in the high-level organisational patterns of the resulting networks.

The application of GNMs is in its relative infancy, but the main lessons are as follows: Biological networks can be simulated by connecting nodes with similar connectivity profiles—a principle called “homophily”—embedded within a physical space. This is consistent across multiple spatial scales, species, and developmental timepoints, including human cerebral organoids (Akarca et al., 2022), mice (Carozza et al., 2022), and non-invasive neuroimaging of child and adult brains (Akarca et al., 2021;Betzel et al., 2016). Individual differences in the trade-off between wiring cost and homophily have predictive validity across different cognitive domains, paradigms, and psychopathology: they differentially predict cognition in neurodevelopmentally diverse children (Akarca et al., 2021), and adaptive stress responses in early life adversity paradigms (Carozza et al., 2022).

To summarise so far, human brains have a characteristic high-level topological organisation, which can be simulated by trading off the cost of forming connections against the “value” they bring to the network. But what underlying factors might shape this organisation or the trade-off that produces it? The human brain is an energetically costly organ. As such, to remain adaptive, brain evolution may force a continuous trade-off between anatomical constraints, the “cost” of wiring, and the adaptive “value” of new connections (Bullmore & Sporns, 2012). In parallel, humans have faster evolutionary rates of brain expansion relative to other non-human primates (seeDeCasien et al., 2022), anchored by relatively evolutionarily preserved unimodal cortices to the greatest evolutionary change within higher-order default-mode networks (Xu et al., 2020). This is thought to, at least in part, shape the higher-order cognitive abilities that distinguish humans from other animals (Xu et al., 2020). One possibility is that the genes associated with cognitive ability are also associated with the topological organisation of the brain and in turn the economic trade-offs needed to simulate that organisation. Indeed, the genetic basis of structural brain connectivity does overlap with that of cognitive ability (Savage et al., 2018;Sniekers et al., 2017).

Furthermore, as shown by genome-wide association studies (GWAS) of tractography-derived white matter connectivity profiles, the topological organisation of structural connectivity networks is reflected by genetic architecture (Sha et al., 2023;Wainberg et al., 2024). Specifically, genes linked to the white matter connectome are enriched for myelination, neural cell proliferation, and cytoskeletal reorganisation (Wainberg et al., 2024), with the strongest enrichment for genes highly expressed during early-to-late prenatal development and within astrocytes (Sha et al., 2023). Alongside structural connectivity itself being polygenic, and with a many-to-one pleiotropic mapping with genetic variants, it also overlaps with polygenic predispositions to neurodevelopmental conditions and cognitive characteristics, including autism and educational attainment (Sha et al., 2023;Wainberg et al., 2024). Recent analyses of the Allen Human Brain Atlas (AHBA;Hawrylycz et al., 2012) have demonstrated enrichment of the third principal gradient of cortical gene expression with both white matter development and common genetic variation linked to intelligence and cognition (Dear et al., 2024).

One approach to assess individual variability in the genetic basis of complex phenotypic traits, such as cognitive ability, is to use polygenic scores (PGSs). A PGS measures an individual’s genetic predisposition towards an outcome by summing each single nucleotide polymorphism (SNP; changes in single DNA nucleotides within a gene), weighted by genome-wide association studies (GWAS) effect sizes (Choi et al., 2020), and require substantially smaller sample sizes than GWAS studies. We focus on PGSs for cognitive ability for the following reasons. First, within the Adolescence and Brain Cognitive Development (ABCD) study, the PGS for cognitive ability is more highly powered than PGSs for psychiatric conditions overlapping with structural connectivity (Vedechkina, 2024), such as anxiety and attention deficit hyperactivity disorder (1.22% variance explained), depression (0.57% variance explained), and psychosis (0.19% variance explained). This may be in part due to the large number of loci (205) linked to intelligence identified in the GWAS from which we developed our PGS (Savage et al., 2018). The second reason is theory driven: a trade-off between wiring cost and topological efficiency, such as that conceptualised by generative network models, is likely to shape key organisational properties of networks (e.g.,Carozza et al., 2023), and some of those properties, like local or global efficiency, have been linked with human intelligence (Barbey, 2018). Third, the PGS for cognitive ability is more easily interpretable and operationalised than for some other constructs. For example, whilst the PGS for cognitive ability is derived from standardised cognitive assessments, the PGS for “educational attainment” is simply based on the number of years in education, and thus it is unclear what that PGS captures (T. Smith, 2023). By linking variability in PGSs for cognitive ability with a generative model reflecting the competing metabolic and topological evolutionary constraints on structural connectivity, alongside network properties, we begin to decipher how and why common genetic influences shape brain organisation.

The aim of the current study was to combine these ingredients to test whether common genetic variants linked to cognition are associated with the high-level organisational characteristics of networks, and changes in the economic trade-offs needed to simulate those organisational characteristics. We first compared different GNMs variants (Betzel et al., 2016) to determine the best-fitting model, before fitting that model to each individual participant. Next, we used PGSs for cognitive ability to test whether and how common genetic variants are associated with measures of network organisation, and the individual generative parameters within the model. Finally, we tested the overlap between the functional role of the genes that contribute to those PGSs and regional AHBA gene expression data (Seidlitz et al., 2020) using a comparative pathway enrichment analysis.

## 2 Materials and Methods

Following a description of the participants, we outline our multiple different data processing and analysis steps. Readers might find it helpful to group these steps into four broad categories, which we will work through in sequence: (i) building connectomes, (ii) genomic analysis, (iii) generative network modelling, and (iv) gene expression analysis (seeFig. 1).

**Fig. 1.**
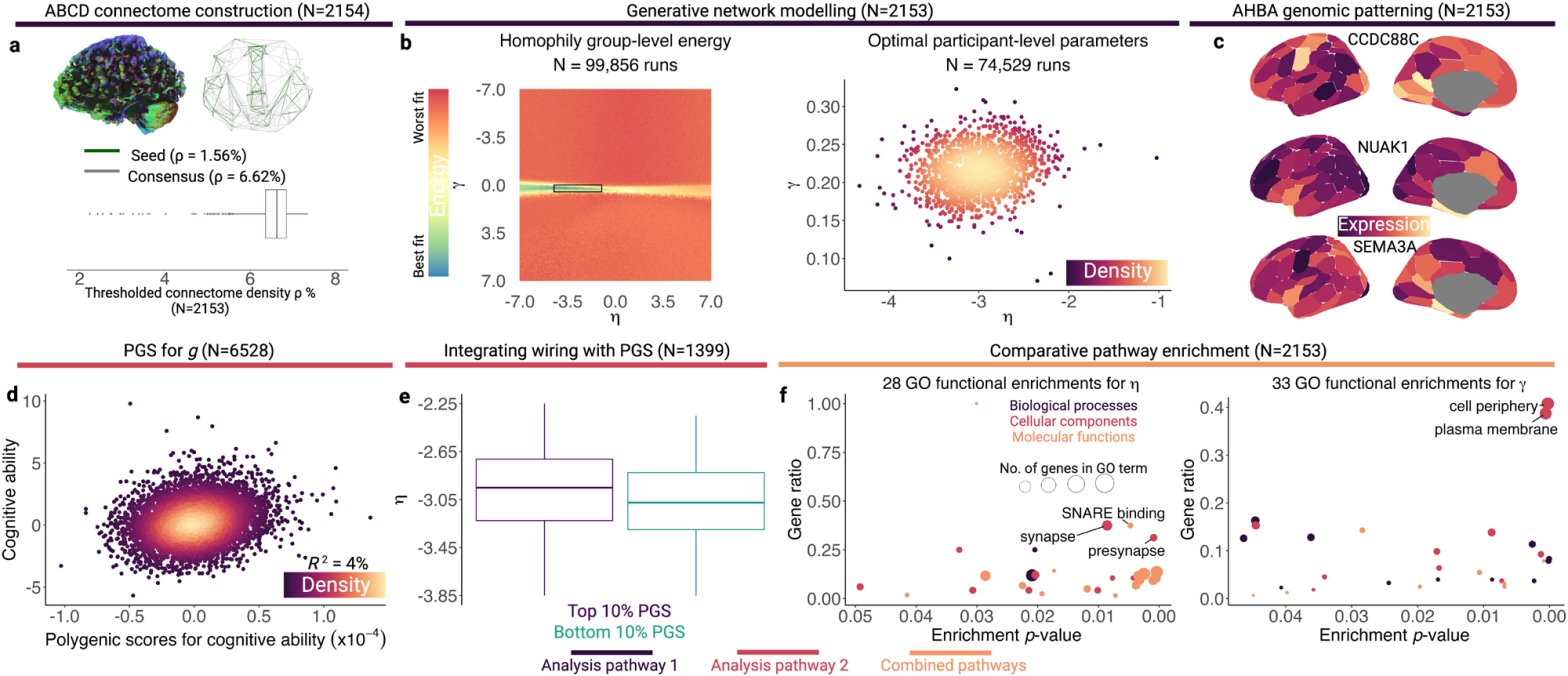
Analytical workflows of neuroimaging, generative simulation, and transcriptomics. Analysis pathway 1 primarily focuses on generative modelling and genetics from the Allen Human Brain Atlas (AHBA), until the final step. Analysis pathway 2 integrates polygenic scores for cognitive ability with generative network modelling. (a) We applied deterministic tractography to reconstruct structural connectomes for 2,154 participants with high-quality neuroimaging data from the ABCD study. The upper left-hand side plot is a tractogram for a representative participant. The upper right-hand side plot shows the seed network (green) used across all simulations, superimposed onto the group-level consensus connectome (grey), embedded in Euclidean space according to the Schaefer 100-node parcellation (Schaefer et al., 2018). The x-axis is an anterior-posterior view, whilst the y-axis is a left-to-right view. The bottom plot shows the distribution of participant-level connectome densities. (b) The left-hand side plot shows the energy distribution of the broad group-level grid search, with the rectangle bounding the low-energy η and γ estimates subsequently used for participant-level simulations. The right-hand side plot shows the optimal participant-level η and γ estimates (N = 2,153). (c) For each participant (N = 2,153), we conducted a partial least squares analysis linking their parameterised nodal wiring costs and values, separately, to nodal measures of cortical gene expression from the Allen Human Brain Atlas (AHBA). Each sub-plot shows the expression distribution of three genes identified by the literature as being particularly important predictors of the white matter connectome (Wainberg et al., 2024). (d) Using summary statistics from a GWAS identifying 205 loci associated with intelligence (Savage et al., 2018), we calculated a polygenic score (PGS) for general cognitive ability “*g*” across 6,528 ABCD participants with high-quality genomic data. (e) To link generative network parameters, genetics, and network organisation, we conducted three sets of analyses. The first involved all participants with PGSs and generative network parameters (N = 1,461), where we contrasted simulated network properties from participants belonging to either the top or bottom 10% of the PGS distribution. The accompanying plot shows that children belonging to the top 10% of the PGS distribution have significantly softer wiring distance penalties. The second involved testing linear associations between generative network model parameters and polygenic scores, following outlier removal (N = 1,399). The third involved simulating how simulated connectome properties change as a function of stochasticity. (f) We conducted single-query and multi-query comparative gene enrichment analyses for PGSs and generative network parameters. The plots show the gene ontology (GO) functional enrichments for parameterised η and γ across all participants with generative network parameters. The gene ratio is the ratio of differentially enriched genes relative to all genes in that ontology. The boxplots in (a, b) and (e) represent the first, second (median), and third quartiles; whiskers represent non-outlier endpoints, whilst crosses represent outliers.

### 2.1 Participants

#### 2.1.1 The ABCD Cohort

In this project we needed sufficient statistical power to detect a possible association between generative network parameters and polygenic scores for cognitive ability. This meant that we needed a dataset with genetic data, diffusion neuroimaging, and gold-standard psychometric data across multiple assessments, for hundreds of participants. This narrows down the available datasets, and the ABCD sample was the only dataset we found that met all these requirements.

We selected participants from the baseline assessment of the ABCD Study Data Release 4.0 (DOI: 10.15154/1523041), comprising 11,876 children (*N*= 5,668 male), aged between 8.92 and 11.08 years (mean = 9.92 ± .63 years), recruited from 21 sites across the United States. The ABCD study was designed to reflect the US population composition (Karcher & Barch, 2021) through race (52.4% White, 17.3% Hispanic, 15.1% Black, 2.1% Asian, and 13.2% Other), and socio-economic status (SES) distribution, including parental working status (69.6% Working, 4.3% Looking for Work, 6% Retired, 17.4% Stay-At-Home Parents, and 8.1% Other), highest parental educational attainment (6.6% less than 12^th^Grade, 10.6% high school graduates, 16.5% with some college experience, 13.0% with an associate’s degree, 28.1% with a bachelor’s degree, 22.0% with a master’s degree, and 3.2% with a doctoral degree), and income-to-needs ratio (INR) (M = 3.36 ± 2.61). Ethical approval for most recruitment sites was obtained from the central Institutional Review Board (IRB) at the University of California, with the remaining sites granted local IRB approval (Auchter et al., 2018).

#### 2.1.2 Participant stratification

To reduce the computational burden associated with reconstructing connectomes and fitting generative models to almost 11,000 children from ABCD with neuroimaging data, we selected a stratified neuroimaging subset of 2,193 children with high-quality T1-weighted (T1w), MRI, rsfMRI, and single-run DWI data. Sample sizes at each stage are shown inFigure 1. For our neuroimaging, we selected a stratified subset of 2,193 genotyped children who had high-quality T1-weighted (T1w) MRI, rsfMRI, and single-run DWI data. To ensure that our subsample was representative of the larger ABCD cohort, we stratified based on 3 variables: general cognitive ability, measured by the NIH Toolbox (NIH-TB) Composite Age-Corrected Score, and binned into 4 groups (32–88, 88–100, 100–112, 112–221); age at time of MRI scans (months), binned into 2 groups (107–119, 119–133 months); and parent-reported sex (2 levels—male and female).

From the stratified sample, we successfully processed data from 2,154 participants across 3 parcellations: Schaefer 100-node 17-network (Schaefer et al., 2018), Brainnetome 246-node (Fan et al., 2016), and Schaefer 400-node 17-network parcellations (Schaefer et al., 2018). We subsequently tested the stratification, relative to the overall sample, with series of bootstrapped Kolmogorov–Smirnov tests, across a series of demographic variables. The distributions of the successfully processed participants did not differ from the larger ABCD sample in terms of age [*D*(2154) = .016,*p*= .572], NIH-TB-Comp age-corrected scores [*D*(2154) = .012,*p*= .955], or income-to-need ratio [*D*(2154) = .028,*p*= .090]. A 2 x 2 chi-square test of sex (two levels—male or female) and sample (two levels—stratified or wider neuroimaging sample) revealed no significant differences in distributions of sex between the two samples [χ^2^(1, 2154) = .027,*p*= .869]. A 5 x 2 chi-square test of parental working status (five levels—working, looking for work, retired, stay-at-home parent, or other) revealed no significant differences in distributions of parental working status between the two samples [χ^2^(4, 2154) = 2.867,*p*= .580]. However, a 7 x 2 chi-square test of highest parental education (seven levels—below 12^th^grade, high school, partial college, associate’s degree, bachelor’s degree, postgraduate degree, or doctorate) revealed a significant difference in distribution of highest parental education between the two samples [χ^2^(6, 2154) = 19.767,*p*= .003, odds ratio (OR) = 1.3]. Similarly, a 5 x 2 chi-square test of race (five levels—Asian, Black, Hispanic, White, or other) revealed significant differences in the distribution of race between the two samples [χ^2^(4, 2154) = 16.377,*p*= .003, OR = 1.335]. This is because biases exist in who has available neuroimaging data.

#### 2.1.3 Imputation

We coded parents who failed to answer questionnaires or were unsure as missing. We imputed missing values across the seven cognitive subscales (n = 241), NIH-TB-Comp measure (*n*= 397), age (*n*= 75), race (*n*= 194), INR (*n*= 386), sex (*n*= 75), parental education (*n*= 92), and parental working status (*n*= 131) using predictive mean matching through Multivariate Imputation by Chained Equations, implemented using the “mice” package (van Buuren & Groothuis-Oudshoorn, 2011).

### 2.2 Building connectomes

We generated structural connectivity matrices for each participant across three parcellations of varying spatial resolution, as described above, for which we conducted group-level analyses. We will refer to just one parcellation here (the Schaefer 100), but interested readers can find various properties of the other parcellations inSupplementary Table S1. Note that the following sections contain information from the QSIprep boilerplate.

#### 2.2.1 Structural MRI acquisition and pre-processing

We downloaded high-quality fast-track structural MRI and DWI from the ABCD-BIDS Community Collection (https://collection3165.readthedocs.io/en/stable/). T1w images were acquired during a single session across an axial plane, with a multiband gradient echo sequence, 8° flip angle, 256 x 256 matrix size, 1,060 ms inversion time, and 1 mm isotropic resolution (Casey et al., 2018;Hagler et al., 2019). Since the ABCD study is a collaborative effort spanning 21 acquisition sites, participants were scanned using 3 possible 3-Tesla scanners, using the following additional scanner-specific parameters: Siemens Prisma VE11B-C (Repetition Time (TR) = 2,500 ms, Echo Time (TE) = 2.88 ms), with 176 slices, General Electric (GE) MR750 DV25-26 (TR = 2,500 ms, TE = 2 ms) with 208 slices, and Philips Achieva dStream or Ingenia (TR = 6.31 ms, TE = 2.9 ms) with 225 slices. For further details, seeCasey et al. (2018)andHagler et al. (2019).

We processed structural MRI data using QSIprep 0.15.3 (Cieslak et al., 2021), implemented through*Nipype 1.7.0*(Gorgolewski et al., 2011),*Nilearn 0.9.0*(Abraham et al., 2014), and*Dipy 1.4.1*(Garyfallidis et al., 2014). Following the T1w image corrected for intensity non-uniformity using the N4 Bias Field Correction algorithm (Tustison et al., 2010), the image was skull-stripped using The Open Access Series of Imaging Studies (OASIS) as a target, creating a T1w reference image. Volume from the T1w reference and OASIS target were used to non-linear spatially register and normalise the T1w reference to the ICBM 152 nonlinear asymmetrical template version 2009c, as a normative brain template for children aged between 4.5 and 18.5 years old (Fonov et al., 2011). The normalised image was then segmented into cerebrospinal fluid, cortical grey matter, and WM using FMRIB’s Automated Segmentation Tool (ANTs;Zhang et al., 2001).

#### 2.2.2 DWI acquisition

Across all 3 scanners, during a single session 81 DWI slices of size 140 x 140 voxels were acquired with 4 b-shells (500, 1,000, 2,000, 3,000 s/mm^2^) across a total of 96 diffusion directions (b = 500 s/mm^2^, 6 directions; b = 1,000 s/mm^2^, 15 directions; b = 2,000 s/mm^2^, 15 directions; b = 3,000 s/mm^2^, 60 directions), with 1.7 mm isotropic resolution, and MultiBand Acceleration Factor 3 (Casey et al., 2018;Hagler et al., 2019). In addition, the following scanner-specific parameters were used: Siemens (TR = 4,100 ms, TE = 88 ms, 90° flip angle), GE (TR = 4,100 ms, TE = 81.9 ms, 77° flip angle), and Philips (TR = 5,300 ms, TE = 89 ms, 78° flip angle).

Using QSIprep 0.15.3, DWI data underwent resampling to T1w space, intensity normalisation through being scaled by b = 0 means, linear co-registration to T1w using ANTs, denoising, and susceptibility distortion (Eddy currents) correction using FSL TOPUP (Andersson et al., 2003;S. M. Smith et al., 2004). The processed DWI data were reconstructed using DSI Studio, which uses non-parametric model-based approaches to fit a multi-tensor model, with generalised q-sampling imaging orientation distribution functions (ODFs) (Yeh et al., 2010) estimated with mean diffusion distance ratio of 1.25. The DWI data then underwent deterministic tractography (Yeh et al., 2013) with 5 million randomly seeded streamlines, between 30 and 250 mm in length, and 1 mm step size. We chose deterministic tractography based on evidence of more accurate connectome reconstructions and lower false-positive rates compared with probabilistic tractography (Sarwar et al., 2019). Note, however, that deterministic tractography does not provide an estimate of confidence of tract orientation, unlike probabilistic tractography.

#### 2.2.3 Connectome construction

We considered structural connectivity as the number of streamlines terminating at each node, yielding a*nroi*x*nroi*matrix per participant, where*nroi*denotes the number of regions of interest in each of the three parcellations investigated. We then averaged across SC for all participants for each parcellation, yielding a single*nroi*x*nroi*matrix. We conducted four thresholding procedures across all parcellations and provide the mean and distribution of densities at each stage for the Schaefer 100-node parcellation. Before thresholding and after removing self-connections, the mean density across 2,154 participants was 12.85% (*SD*= 1.46%). First, to remove spurious connections, we retained connections common to at least 60% of participants, resulting in a mean density of 6.61% (*SD*= .51%). Second, using these connectomes, we applied a consensus-based distance-dependent threshold across all participants, which has been shown to preserve key statistical network properties and edge length distributions (Betzel et al., 2019), yielding a single binary consensus network across all participants, with density of 6.69%. This was the group-level modelling target, which we used to evaluate our different model classes. This relative sparsity is required for generative modelling as computational power needed to run the simulations scales with connection number, and is comparable with previous studies (Betzel et al., 2016). Third, we binarised the participant-level connectomes by thresholding at 27 streamlines (Akarca et al., 2021). These were the participant-level modelling targets, with mean density of 6.55% and standard deviation of .50% (seeFig. 1a). Fourth, to create a seed network for all simulations (i.e., a basic common starting point across the group-level and participant-level simulations), we selected connections present in at least 95% of participants from the participant-level generative modelling targets, producing a seed network with density of 1.58%. After removing one participant whose density (ρ = .02%) suggested incorrect connectome reconstruction, this meant that the generative network model added 66 connections to simulate the least sparse individual-level target connectome, 572 connections to the densest individual-level target, and a modal number of 512 connections.

### 2.3 Genomic analyses

#### 2.3.1 Genomics acquisition and quality control

Genotyping data were provided by the ABCD study, where SNPs from saliva and whole blood samples were extracted and processed by Rutgers RUCDR, and assayed using the Affymetrix NIDA SmokeScreen Array, made up of 646,247 markers (Baurley et al., 2016;Uban et al., 2018). Genotyping quality control was conducted by the ABCD DAIRC Team, using recommendations from the Ricopili pipeline which, in brief, consisted of pre-imputation quality control to ensure consistency of sample identifiers, PCA to identify duplicates and population structure, imputation to a reference panel to maximise SNPs, alongside post-imputation clumping of genome-wide significant SNPs and incorporation of covariates (seeLam et al., 2020;Uban et al., 2018). Together, 11,099 participants with 516,598 SNPs passed ABCD QC (see ABCD Study 4.0 Release Notes).

Our discovery GWAS dataset was provided bySavage et al. (2018), which consisted of 267,867, principally European, participants, using the NCBI GRCh37 Genome Build, spanning 9,295,118 SNPs. As part of QC for the discovery dataset, we removed SNPs with minor allele frequency (MAF) less than .10, imputation information scores less than .80, ambiguous and duplicated SNPs with multiple or missing bases, resulting in 6,101,992 SNPs.

We selected genotyped participants from the ABCD Baseline with complete demographic data and excluded those with axiom plate 461 known to be problematic, generating target dataset of N = 10,979. Note that we downloaded genetic data from the ABCD Study Data Release 3.0, which was automatically provided by the most recent data release, due to no differences between releases. Using PLINK 1.9 (Chang et al., 2015;Purcell et al., 2007;Purcell & Chang, 2019), we performed additional QC on the target dataset, detailed elsewhere (Choi et al., 2020). In total, 8,320 SNPs had incorrectly formatted unique identifiers, therefore, associated allelic data were obtained from the Ensembl Gene Browser (GRCh37), and non-ambiguous, unique matches used to update the binary PLINK files. Next, we removed SNPs with MAF <.10, Hardy–Weinberg Equilibrium <1e^-6^, genotyping rate >.99, and missingness rate <.01, and then pruned the resulting SNPs with linkage disequilibrium variance (LD r^2^) higher than .25 in steps of 50 variants across a window size of 200 variants. Further, we removed participants with heterozygosity F-coefficients greater than 3 standard deviations above or below the mean. We retained effect alleles which did not require strand flipping, recoding, or whose bases were not complementary between the base and target datasets. Finally, we removed participants with discordant sex information and relatedness >.125. Together, 6,528 participants (52.88% male, 81.53% European), aged between 8.92 and 11.08 years old (Mean = 9.93 ± .62 years), with 197,798 SNPs passed QC.

#### 2.3.2 Polygenic score calculation

A PGS is the sum of “risk” alleles, in this case predisposing children to high cognitive ability in ABCD, weighted by the magnitude of their association with a phenotype from a GWAS (Choi et al., 2020;Savage et al., 2018). Our GWAS had identified 205 loci linked to intelligence in an independent sample of over a quarter of a million adults. We tested the association between the PGS and the phenotype of interest,*g*, using a linear regression, accounting for sex and six principal components of population structure, separately for European and non-European participants. This produced an adjusted*R^2^*measure, which is the variance in*g*the PGS accounts for. The distribution of polygenic scores is shown inFigure 1d.

Since ancestry is a major confounding variable in PGSs (seeChoi et al., 2020), we first inferred the underlying population structure using*k*= 4 ancestral groups in fastStructure (Raj et al., 2014). We found that the optimal*k*ranged between 1 and 4 and, therefore, for simplicity, decided upon*k*= 2 clusters. Following prior ABCD studies (Loughnan et al., 2021), we stratified our analyses into those of over 90% European ancestry (N = 7,500) and classified the remaining participants (N = 3,479) as Non-European. We performed the PGS in PLINK 1.9. We clumped all SNPs and retained those with an LD*r^2^*> .1 and calculated the PGS against 7*p*-value thresholds for SNP inclusion (.001, .05, .1, .2, .3, .4, .5). Finally, to account for population structure, we adjusted our PGS by including six population principal components as covariates. This resulted in PGSs calculated for 6,528 participants, 1,486 of which overlapped with the stratified sample, and 1,461 of which had successfully processed structural connectomes and GNMs.

For our target phenotype, rather than restricting our analyses to specific cognitive subscales, we extracted a general “g” factor through principal component analysis (PCA) of the seven NIH-TB subscales using the FactoMineR package (Lê et al., 2008). We took the first principal component (PC) across seven different tasks from the NIH toolbox, which had been administered to all participants, spanning attention (Eriksen & Eriksen, 1974;Rueda et al., 2004;Weintraub et al., 2013), set shifting (Zelazo, 2006), working memory (Tulsky et al., 2014), episodic memory (Dikmen et al., 2014), language (Gershon et al., 2013;Woollams et al., 2011), and processing speed (Carlozzi et al., 2015). A 5-factor solution accounted for 85.04% of variance in the cognitive battery. The first PC—hereafter labelled “*g”*—captured 37.76% of variance in cognitive scores. Crucially, each task contributes roughly equally to this variance (Picture Vocabulary, 15.52%; Flanker, 14.16%; List Sorting, 16.55%; Dimensional Card Sorting, 16.57%; Pattern Comparison, 11.49%; Picture Sequence Memory, 10.64%; Oral Reading Recognition, 15.06%), as we would expect with a*g*factor (seeDeary et al., 2010).

### 2.4 Generative network modelling

GNMs simulate connectivity from a sparse seed network by iteratively calculating the probability of each pair of nodes being connected until the number of connections in the synthetic connectome matches that of the empirical connectome (Betzel et al., 2016;Kaiser & Hilgetag, 2004;Vértes et al., 2012). A sparse seed ensures that the trajectories of the simulations stem from a reasonable starting path, whilst also not overly restricting the scope of the simulations.

$$P_{i,j}\propto {\left(D_{i,j}\right)}^{\eta }\;{\left(K_{i,j}\right)}^{\gamma }.$$

The wiring equation (above) has a geometric and non-geometric component, the product of which is proportional to the probability a connection forms between any two nodes*i*and*j*. The geometric component represents the physical distance between each pair of nodes, such as the Euclidean distance*D_i,j_.*The non-geometric component*K_i,j_*represents the potential topological value of connections between each pair of nodes. This can be conceptualised across four classes: clustering (tendency of nodes to group together), degree (number of connections), geometric (where*K_i,j_*= 1), and homophilic (similarity in nodal connectivity profiles). Within the clustering and degree classes, topological value is driven by either the minimum, maximum, mean, product, or difference between nodal properties, constituting five rules for each class. Within the homophilic class are two closely related varieties: neighbours, representing the similarity in connectivity profiles between two nodes, and the matching index, which is the normalised equivalent. The mathematical similarity of the homophilic models is reflected in their similar performance across the top-performing simulations only, but not across poorly fit simulations (Supplementary Table S4). The current study considered five generative models: the geometric model, the two homophilic models, and the top-performing clustering (average) and degree (average) models based on the literature (Akarca et al., 2021; seeBetzel et al., 2016for model specifications).

In terms of how this unfolds practically, we first calculate the Euclidean distance between all pairs of nodes*D_i,j_*, which remains fixed across simulations, and raise it to the power η, which varies across simulations. We then calculate the topological value of connections between all pairs of nodes*K_i,j_*according to the non-geometric part of the wiring equation and raise it to the power γ. Generative rules were specified based on a power-law as prior work has shown this to be superior to an exponential relationship (Vértes et al., 2012). For the initial simulation, ∈ = 10^6^is added to*K_i,j_*to ensure that*P_i,j_*is always defined. We multiply (*K_i,j_*)^γ^and (*D_i,j_*)^η^, exclude already connected pairs, and extract the non-zero elements as the wiring probabilities. The wiring value term*K_i,j_*is updated dynamically within each simulation as the topological properties of the simulated network vary with each new connection. This continues iteratively until the simulated network has the same number of connections as the empirical network. By collecting (*D_i,j_*)^η^and (*K_i,j_*)^γ^at each pair of nodes and averaging across iterations, using the participants’ optimal η-γ trade-off, we produce the parameterised nodal wiring costs and values presented inFigure 4b and c, respectively.

We performed two sets of analyses—at the group level, and at the participant level. At a group level we wanted to establish which of the five wiring rules described above best accounts for the wiring properties of the group connectome. We performed a grid search using 100,000 equally spaced random η [-7 ≤ η ≤ 7] and γ [-7 ≤ γ ≤ 7] combinations (99,856 unique combinations). We selected the η and γ boundaries as in a recent GNM paper (Akarca et al., 2021). We performed a grid search using 100,000 equally spaced η [-7 ≤ η ≤ 7] and γ [-7 ≤ γ ≤ 7] combinations (99,856 unique combinations). Since wiring cost constrains the structural connectome (Bullmore & Sporns, 2012), we expect that the optimal η estimates will be negative, representing an increased penalty on long-distance connections. However, we examined the full range of possible η because a positive bias towards long-distance connections is theoretically possible depending on the wiring value term, such as average degree and homophily.

To select the best-fitting model, we used three criteria. The first, and most common way to evaluate generative model performance, is through an energy function (Akarca et al., 2021,^2021^;Arnatkeviciute et al., 2021;Betzel et al., 2016;Zhang et al., 2021), as shown below. This is a global measure of model fit.

$$E\;\;=\;\;\max\left(KS_{k},\;\;KS_{c},\;\;KS_{b},\;\;KS_{e}\right).$$

A Kolmogorov–Smirnov (KS) test assesses whether the distribution of four statistics significantly differs between the synthetic and observed connectomes: degree (*k*), clustering (*c*), betweenness-centrality (*b*), and edge length (*e*). The largest KS-statistic is the model energy (Betzel et al., 2016). Therefore, the model class with the smallest energy, hence smallest dissimilarity, was deemed the best fit. However, whilst energy is a good global measure of fit, we also assessed the ability of the synthetic connectomes to capture distributions of*local nodal*properties. To examine the ability of synthetic connectomes to recapitulate the local nodal properties of empirical connectomes, rather than the global measure of fit in energy (Akarca et al., 2022;Carozza et al., 2022), we calculated each network’s topological fingerprint (TF). This is a 6 x 6 correlation matrix of 6 local network statistics, showing the relationships between these local properties across nodes: degree, clustering, betweenness-centrality, edge length, local efficiency, and eigenvector centrality. The Euclidean norm of the difference between the topological fingerprint of the empirical network (TFempirical) and the synthetic network (TFsynthetic) produces a single TF dissimilarity value.

$$TF_{dissimilarity}=\sqrt{\sum_{i}\sum_{j}{\left(TF_{empirical_{i,j}}\;\;-\;\;TF_{synthetic_{i,j}}\right)}^{2}}.$$

A complementary metric of model fit is spatial similarity, which is a correlation of nodal degree between synthetic and empirical connectomes, and tests for the explicit spatial overlap across regions. Topological fingerprints and spatial embedding answer slightly different questions (Carozza et al., 2023). That is, the former tests similarities in global organisation across networks, whilst the latter tests the anatomical similarity in placement of nodes. This means that two networks can have similar global topology (low topological dissimilarity) but be embedded in different parts of anatomical space (weak spatial embedding). Unlike the previous two evaluation metrics, this tests whether the spatial patterning of the connections is similar between the observed and simulated connectomes. Again, because of the probabilistic nature of the GNM, we repeated this process 1,000 times for the lowest energy parameter combination for each model.

We ranked each model according to these three criteria and selected the model with the smallest cumulative rank as the best fit. We then took this model forward and fit it to each individual participant. We performed an additional 50,000 simulations for each participant, using η and γ parameter boundaries corresponding to the top 10% lowest energy group simulations (49,729 unique combinations). To explore how GNM terms varied across the simulation, for our individual-level analyses, we collected node-wise parameterised*D_i,:_*and*K_i,:_*terms for each new connection added until the target was reached. Throughout, we used the ggseg R package (Mowinckel & Vidal-Piñeiro, 2020) to plot nodal measures on the cortical surface, and the ggplot2 R package (Wickham, 2016) to visualise correlations of observed and simulated network statistics.

#### 2.4.1 Comparing networks and model parameters across participants

Having built the individual participants’ connectomes, selected the best-fitting model, fit that model to each participant, and established the optimal trade-off for each simulation, we next tested whether those optimal parameters differ across participants, the distribution of which are shown inFigure 1e. We removed participants whose modelling parameter values were more than 2 standard deviations from the mean, resulting in a final sample of 1,399 children. Our primary way of comparing participants was also the simplest—we identified participants with the top and bottom 10% PGS for cognitive ability and compared their modelled η and γ parameters with independent sample t-tests. In essence, is someone’s genetic propensity for cognitive ability related to the economic trade-off needed to simulate their network? This comparison of extreme groups is admittedly simple, but it means we can then subsequently simulate networks with these group average parameter combinations, to test in more detail how differences in model parameters directly shape the resulting simulated networks. Further, the comparison of extreme groups across 1,000 simulations also allows us to test*variability*across simulations, and establish whether this is systematic and informative, rather than just noise. Based upon previous studies, if you soften the wiring parameters, you create a simulation process that is systematically more stochastic, introducing types of variability depending upon the parameters that are softened (Carozza et al., 2023). We also examined continuous relationships between model parameters and PGSs using a series of generalised linear models and conducted a series of experiments to assess the impact of stochasticity on simulated network properties.

### 2.5 Gene expression analysis

#### 2.5.1 Allen Human Brain Atlas microarray processing

We used the Allen Human Brain Atlas microarray data as measures of regional brain gene expression (https://human.brain-map.org/). This dataset contains transcriptomic data for 6 donors (*N*= 5 male) aged between 24 and 57 years old, across 3 ethnicities (AHBA Case Qualification and Donor Profile Technical White Paper), spanning over 62,000 probes and 26,000 genes, with approximately 500 samples collected per hemisphere per donor (AHBA Microarray Survey Technical White Paper). To reduce the influence of methodological factors, AHBA performed additional within-batch quality control, such as adjusting for intensity distribution, RNA quality, batch effects, and dissection method, and between-batch quality control steps, including aligning human brain atlas control and mean expression values (AHBA Microarray Data Normalization Technical White Paper). Further methodological details are available from the AHBA Technical White Papers listed above (https://help.brain-map.org/display/humanbrain/Documentation).

Whilst we initially intended to use the Schaefer 400-node parcellation for gene expression and neuroimaging analyses, to maximise spatial resolution, we found that the distribution of the number of samples provided for each AHBA region [mean = 8.94, median = 6.50, mode = 1.00] and the number of donors providing samples [mean = 3.39, median = 2.00, mode = 2.00] was positively skewed, suggesting that a lower-resolution parcellation may offer better spatial coverage. The degree of bilateral missingness decreased from 13.00% for the Schaefer 400-node parcellation to 3.66% for the Brainnetome 246-node parcellation, to 0% for the Schaefer 100-node parcellation, with RNA-sequencing probe selection, 0.5 intensity-based filtering, and 2 mm region assignment distance threshold. Considering that missingness increases with higher parcellation spatial resolution, and foreshadowing integrating generative network modelling results with AHBA genomics, we selected the Schaefer 100-node parcellation as this had no missing regional micro-array data. A sparser parcellation also reduces the computational cost of the modelling.

Although within-batch and between-batch normalisation was conducted by the AHBA, additional variability can occur through researcher-led decisions about assigning AHBA gene expression values to a parcellation. Therefore, we followed the best-practice guidance ofArnatkevic̆iūtė et al. (2019)when parcellating the AHBA microarray data, and used the*abagen*toolbox (Arnatkevic̆iūtė et al., 2019;Hawrylycz et al., 2012;Markello et al., 2021) in Python 3.10 (seeSupplementary Methodsfor details of AHBA processing).

#### 2.5.2 Gene expression and model parameters

To explore the relationship between AHBA gene expression and nodal parameters from the GNM, we conducted two PLSs for each participant. For each participant, the predictor variables were the left-hemisphere regional AHBA gene expression values, with dimensions 50 (regions) x 12,431 (genes). The response variable in PLS1 was nodal parameterised wiring*costs*(1 x 50 matrix) for that participant, whilst the response variable in PLS2 was nodal parameterised wiring*value*(1 x 50 matrix) for that participant. For each PLS and participant, we conducted 5,000 permutations to derive a null distribution of the loadings of each gene onto each of 3 PLS components (as inAkarca et al., 2021).

For each AHBA gene, PLS, and participant, we conducted 10,000 permutations to generate a permuted*p*-value. We extracted AHBA genes which significantly (*p_perm_*< .05) predicted either parameterised nodal wiring costs or value across all participants, averaged their nodal loadings onto the first PLS latent variable across participants, and ordered the genes by descending loading. This produced ranked lists of 951 AHBA genes for parameterised nodal wiring cost, and 561 for parameterised nodal wiring value (Akarca et al., 2021;Whitaker et al., 2016).

#### 2.5.3 Gene ontology and pathway enrichment analyses

To examine the functional processes implicated in η, γ, and PGSs for cognitive ability separately, we conducted a series of single-query enrichment tests (seeSupplementary Methods). Rather than qualitatively describing similarities between the three single-query enrichment tests, the multi-query comparative enrichment allows us to quantify the overlap in functional enrichment of the generative modelling parameters and PGS statistically, producing a set of*p*-values for each gene list and the size of each intersection. The relative contribution of each gene list to enriched pathways and ontological groups is an additional piece of information provided by the comparative enrichment analysis. The first list was ranked by decreasing X loading onto the first extracted PLS component of parameterised nodal wiring costs regressed onto the AHBA gene expression matrix across the left hemisphere. The second list was ranked according to the equivalent analysis for parameterised nodal wiring value. The third list contained the 76,745 SNPs which were included in the cognitive ability PGS. We ranked each SNP by decreasing absolute contribution β to the PGS. We conducted pathway enrichment analyses for each of the three gene lists, alongside multi-query ranked comparative enrichment tests, using g:Profiler through the gProfiler2 v0.2.1 R package (Kolberg et al., 2020), using the Gene Ontology database (v e107_eg54_p17_bf42210;Ashburner et al., 2000;The Gene Ontology Consortium, 2021). The results were visualised using EnrichmentMap 3.3 (Merico et al., 2010) in Cytoscape v3.9.1 (Shannon et al., 2003), and annotated using the Cytoscape AutoAnnotate plugin (Kucera et al., 2016) through the Community Cluster algorithm from clusterMaker2 (Morris et al., 2011). The Cytoscape session used to visualise these networks is available in the GitHub repository accompanying this paper.

## 3 Results

Mirroring the structure of the Materials and Methods section, we will (i) first describe the properties of the connectomes we reconstructed; (ii) report on the success of the PGS calculation; (iii) outline the group and individual generative network modelling; (iv) compare the model parameters across participants; (v) triangulate PGSs, GNM parameters, and empirical connectome properties; and (vi) report the comparative pathway enrichment analysis. Enriched gene ontologies for parameterised wiring costs and values, respectively, are shown inFigure 1f.

### 3.1 Structural connectomes

**Fig. 2.**
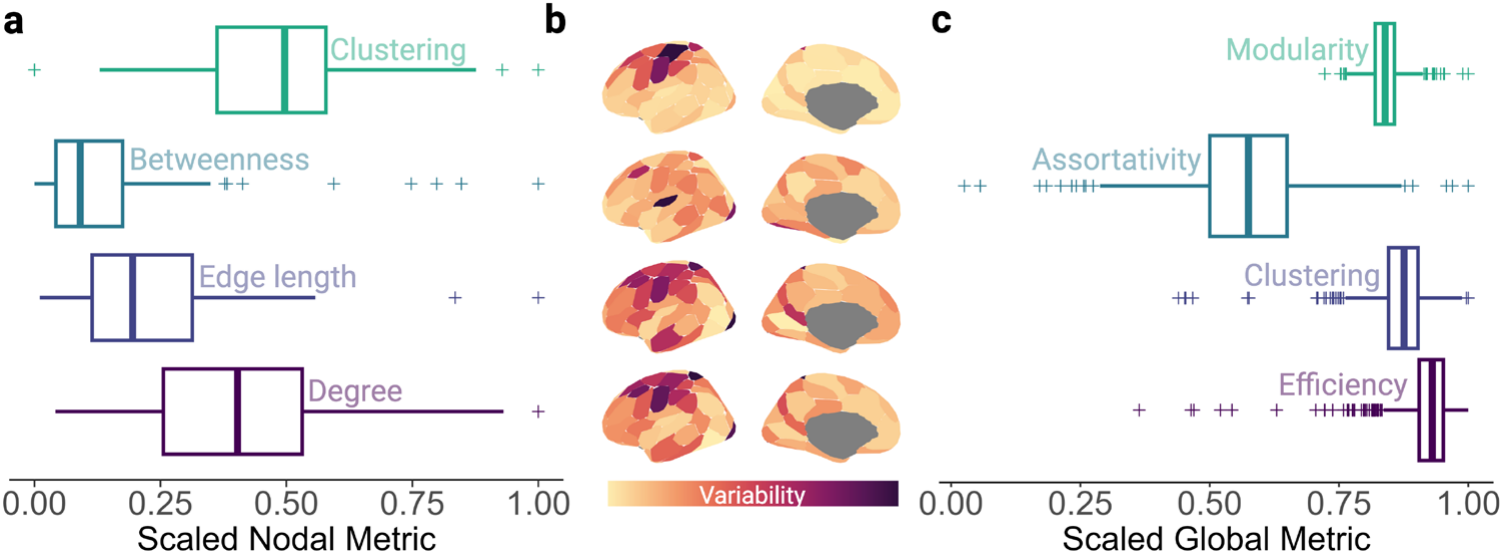
Distributions of local and global graph theory metrics for empirical connectomes. (a) The distributions of four nodal-level metrics used to evaluate simulation performance in subsequent analyses, averaged across participants. Clustering quantifies the proportion of nodal neighbours which are neighbours of each other (Watts & Strogatz, 1998); betweenness-centrality measures the proportion of edges between pairs of nodes which are the shortest paths (Kintali, 2008); edge length is the inter-nodal Euclidean distance; whilst degree is the number of connections a node has. (b) Corresponding variability of nodal metrics, quantified by the coefficient of variation across participants, is patterned spatially (left hemisphere visualised for ease). (c) The distribution of global graph theory metrics. The modularity coefficient quantifies how well a network can be divided into sub-networks that maximise within-network edges and minimise between-network edges (Newman, 2006); assortativity measures how nodes with a similar number of connections wire together (Newman, 2002); global clustering is nodal clustering averaged across nodes; whilst global efficiency is the mean of the most direct paths between each pair of nodes (Latora & Marchiori, 2001). Within (a) and (c), the middle line of the box plot represents the median, flanked by the lower and upper quartiles, respectively. The whiskers represent non-outlier minima and maxima, whilst crosses represent outliers.

We performed tractography and constructed connectomes for each participant, prior to generative modelling. However, before proceeding with that modelling, we first explored the characteristics of the empirical thresholded connectomes (mathematical equations for graph theory measures are provided in theSupplementary Methods). Across nodes, averaged across participants, the distributions of degree and clustering were approximately symmetrical, with skewness values of .49 and -.30, respectively (Fig. 2a). The distribution of edge length was highly positively skewed (1.71), indicating a higher proportion of shorter low-cost edges, with a few long-range high-cost edges. Betweenness-centrality, which measures the proportion of paths passing through a node which are its shortest paths, was also highly positively skewed (2.51). This suggests that for most nodes, the proportion of paths which are the shortest and most direct paths is relatively small. Readers can find these statistics for other parcellations inSupplementary Table S1. To quantify nodal variability of these metrics across participants, we calculated the coefficient of variation (Fig. 2b). For degree and edge length, we observed an axis of between-subject organisational variability extending from maximum variability within the right stomato-motor cortex, to minimal variability within bilateral visual peripheral networks. Minimal variability in betweenness-centrality was observed in bilateral limbic and dorsal attention networks, and maximal variability within bilateral stomato-motor networks. Finally, we observed an axis of maximal clustering variability within left somato-motor and dorsal attention networks, and minimal variability within bilateral visual peripheral networks. We then examined the distribution of global graph theory metrics across participants (Fig. 2c). Whilst the distributions of assortativity (-.17) and modularity (.32) were approximately symmetrical, global efficiency and clustering were strongly negatively skewed, with skewness values of -3.83 and -2.07, respectively. This suggests that relatively few participants had connectomes with low efficiency or clustering.

### 3.2 PGSs for cognitive ability

The PGSs were a significant positive predictor of PC1 loadings (the*g*factor) across both European (*t*= 14.38,*p*= 5.12 x 10^-46^,β= 13024.31,*R^2^*= .04) and non-European (*t*= 4.56,*p*= 5.58 x 10^-6^,β= 12194.43,*R^2^*= .02) subsets with clumping inclusion thresholds of*p*= .10 and*p*= .20, respectively. These loadings are broadly in line with multiple other PGSs of cognitive and behavioural phenotypes and indicate that we have accurately captured the established common genetic variants that are associated with cognitive ability in the wider population. Further statistical details on polygenic model specification and performance at different clumping thresholds can be foundSupplementary Tables S2 and S3, respectively.

### 3.3 Homophilic principles recapitulate childhood structural connectivity

**Fig. 3.**
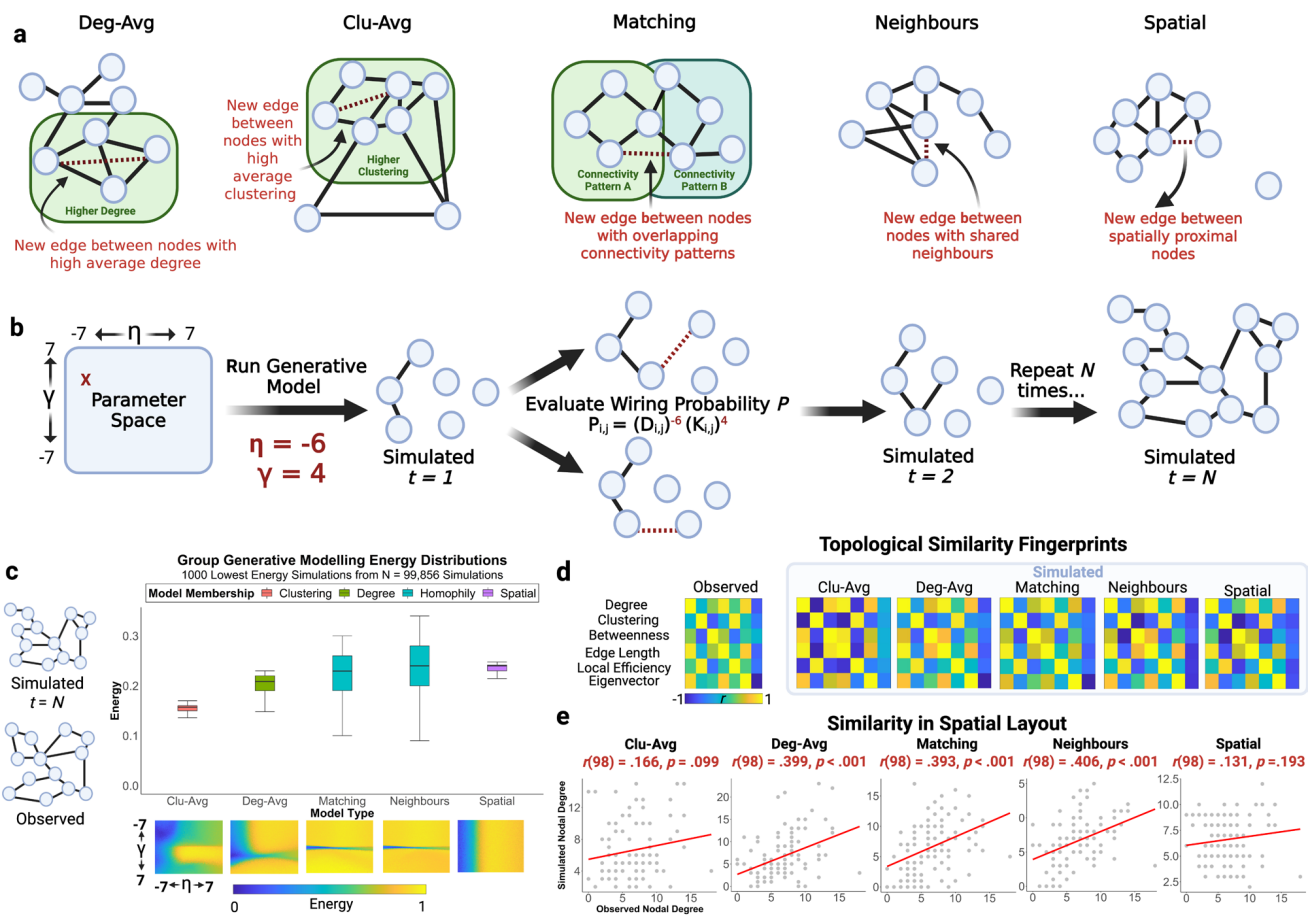
Generative network modelling protocol and evaluation. (a) Five generative network modelling rules were evaluated. The deg-avg (degree average) model adds edges to nodes with high average degrees. The clu-avg (clustering average) model adds edges to nodes with high clustering coefficients. The homophily matching model adds edges to nodes with a high matching index, or normalised similarity in connectivity profiles. The homophily neighbour model adds edges to nodes sharing similar neighbours. The spatial model adds edges to proximal nodes. (b) A random wiring cost (η) and wiring value (γ) combination from a parameter grid search. This combination is used to run all five generative models, with N iterations. At each iteration, the wiring probability between each connected and unconnected node is computed. (c) The simulated connectome is compared with the observed connectome using three metrics. The first is model energy (middle), equal to the largest Kolmogorov–Smirnov statistic testing differences in distributions of edge length, clustering, betweenness-centrality, and edge length. Distributions are shown for five models across four model classes. Corresponding energy landscapes are visualised. The homophily model energy landscapes show convergence on a small parameter window, despite having a large energy range, in contrast to the other models. (d) The second metric is topological similarity, which assesses the ability of the simulated connectome to recapitulate the local distribution of nodal properties in the observed connectome. The fingerprint plotted is representative of the overall statistical trends for each model across all parameter combinations and 1,000 simulations of the lowest energy parameter combinations. (e) The third metric is similarity in spatial layout, equal to the correlation between observed and simulated nodal degree. Simulated degree representative of the mean Pearson correlation between simulated and observed degree across 1,000 simulations of each wiring rule’s lowest energy parameter combination is plotted.

Next we fit generative network models (GNMs) to the group-level consensus network. To remind the reader, different ways of achieving economical brain organisation are implemented in GNMs through different wiring rules, broadly grouped into four classes (Fig. 3a): degree, clustering, homophily, and spatial. At a group level, we first ran 100,000 simulations for the top-performing model classes based on the literature (Akarca et al., 2021;Betzel et al., 2016). This yielded 99,856 unique η [-7 ≤ η ≤ 7] and γ [-7 ≤ γ ≤ 7] combinations (Fig. 3b). We evaluated the similarity between the simulated and observed connectomes using three different measures of fit (Fig. 3c). The first is energy, equal to the largest dissimilarity between synthetic and observed connectomes in terms of one of four nodal metrics: degree, clustering, betweenness-centrality, and edge length distributions (Betzel et al., 2016). The lower the energy, the better the model fit. The homophily neighbours model achieved the lowest energy (.090), with η as -2.911 and γ as .244, respectively, followed closely by the homophily matching model (.100). Across the best 25 simulations for each model, the 2 homophily models performed best [*F*(4, 124) = 577.11,*p*= 2.48 x 10^-77^], with follow-up post hoc tests confirming that these 2 models performed better than the other model types (all*p*< .001), but equivalent relative to each other (*p*= .69). Despite the homophily models having the smallest energy, they also displayed the largest range for the lowest energy 1,000 simulations of .250 and .200, respectively. This suggests that homophily can produce very low energy simulations, but these simulations are the most sensitive to parameter tuning. As shown in the corresponding energy landscapes (Fig. 3c), the 10% lowest energy simulations for, for instance, the homophily neighbours model corresponded to a small region of the convex optimisation space, bounded by a narrow range of η [-5.489 ≤ η ≤ -1.800] and γ [.111 ≤ γ ≤ .333], respectively. To assess the reliability of homophily as the top-performing class, we examined model energy across different simulation thresholds (top 1, 10, 25, 500, and 1,000 simulations). Within a narrow window in which the best simulations were included (top 1, 10, and 25 simulations), the homophily neighbours class had consistently lower energy (and better fit) than all other classes (seeSupplementary Table S4). This narrow window ensures we compare*like with like*, as a small number of models drive the between-class differences. However, once the window was increased to the top 500 or 1,000 simulations, therefore, including more poorly fit simulations and greater ambiguity in which mix of simulations produced the mean energy, the differences between the quality of the simulations drop.

Energy, whilst the most common measure for fitting GNMs (Akarca et al., 2021;Betzel et al., 2019;Zhang et al., 2021), only tests for similarities in global nodal distributions. Therefore, we also used a second evaluative metric, termed the*topological fingerprint*(Fig. 3d). The models differ significantly on their topological dissimilarity [*F*(4, 4999) = 2774.22,*p*< .001]. Homophily neighbours model is the best performing numerically, although both homophily models do well and equivalently (*p*= .209), relative to both the spatial (*p*< .001) and cluster-average models (*p*< .001). The degree-average model also performs well on this metric, performing significantly better than the homophily matching model (*p*= .004) and equivalently to the homophily neighbours model (*p*= .643).

The third and final evaluative metric (Fig. 3e) is similarity in spatial layout, equal to the correlation between the nodal degree of simulated and observed connectomes. Again, we repeated this calculation 1,000 times for the lowest energy parameter combinations for each model. This measure differed significantly across the models [*F*(4, 4999) = 3466.96,*p*< .001], with the homophily models performing better than all other classes (all*p*< .001). The homophily neighbours variant achieved the highest spatial correlation (mean*r*= .42), which is significantly better than all other models, including the homophily matching variant (all*p*< .001).

In summary, the homophily models perform particularly well across all three metrics. We carried forward the homophily neighbours model for our subsequent analyses because it achieved an equivalent energy, and slightly better topological dissimilarity and spatial correlation, relative to the other homophily variant. Note, these two models are mathematically extremely similar, which would explain why they perform so similarly. The lowest energy η and γ combinations, alongside mean topological dissimilarity and correlations between simulated and observed nodal degrees across 1,000 simulations for the lowest energy parameter combinations in the Schaefer 100-node, Brainnetome 246-node, and Schaefer 400-node parcellations are provided inSupplementary Tables S4, S5, and S6, respectively.

### 3.4 Homophily features negative distance penalty and positive topological term

The next step was to fit the homophily neighbours model to individual participants. To narrow down the parameter window, we used η and γ limits which bounded the top 10% lowest energy simulations from the consensus networks ([-7.000 ≤ η ≤ -.200] and [-.067 ≤ γ ≤ .600]). This yielded 74,529 unique combinations of η and γ, which were used to run simulations for 2,153 participants. From the original sample of 2,154, we removed one participant from the subsequent fitting procedure because of incorrect connectome construction.

**Fig. 4.**
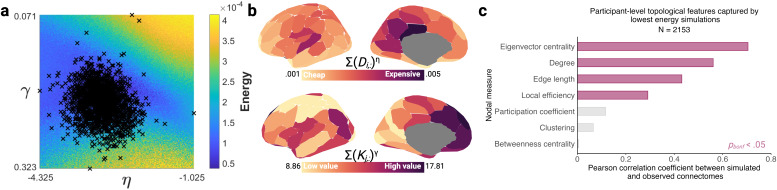
Lowest energy individual homophily neighbour generative network models display regional heterogeneity in parameterised nodal wiring costs and value and recapitulate the distribution of local nodal statistics both included and excluded from the energy equation. (a) Lowest energy η and γ parameter combinations for 2,153 participants, plotted against the associated energy landscape. (b) Averaged across participants, parameterised wiring costs for each node*i*are plotted as the sum of the Euclidean distance with all other nodes, parameterised by η (left hemisphere visualised for ease). Averaged across participants, parameterised wiring values for each node*i*are plotted as the matching-neighbours value term index with all other nodes, parameterised by γ. Details of how parameterised nodal wiring value and costs were calculated are given in the generative network modelling methodology section. (c) Pearson correlations between simulated and observed nodal statistics. We extracted the lowest energy η-γ combination for each of the 2,153 participants and the resultant synthetic connectome. For each synthetic and empirical connectome, we calculated six nodal attributes: degree, edge length, betweenness-centrality, clustering, local efficiency, and eigenvector centrality (seeSupplementary Methodsfor mathematical definitions). This produced an array of dimensions 100 (number of regions) x 2,153 (number of participants) x 6 (number of measures) x 2 (simulated or empirical). For each measure, we averaged across participants to produce two 1 x 100 arrays: one for simulated connectomes and one for empirical connectomes. The Pearson correlation coefficients are visualised, following correction for multiple comparisons using the Bonferroni (*bonf*) method (filled bars represent correlations significant at*p_bonf_*< .05). Note that the correlation coefficient for betweenness-centrality was negative but is plotted as an absolute value for ease.

As shown inFigure 4a, energy for the optimal simulations ranged between .050 and .140 (mean = .071 ± .007). Distance penalties η ranged between -4.325 and -1.025 (mean = -3.007 ± .336). These were all negative and larger in magnitude than the positive topological terms γ, which ranged between .071 and .323 (mean = .215 ± .027). The small standard deviations for optimal η and γ confirmed that the parameter search was successful in a convex optimisation parameter window in the energy landscapes. The distribution of parameterised nodal wiring costs and values, respectively, are visualised inFigure 4b. The simulations recapitulated several features of the observed connectomes (Fig. 4c), such as degree [*r*(98) = .561,*p*< .001] and edge-length [*r*(98) = .431,*p*< .001]. Crucially, they also recapitulated the emergence of local statistics excluded from the energy equation and model selection, such as local efficiency [*r*(98) = .291,*p*= .003] and eigenvector centrality [*r*(98) = .705,*p*< .001], However, the simulations struggled to accurately model the distributions of betweenness-centrality, and clustering, typically under-stepping.

### 3.5 Children with a stronger genetic predisposition for high cognitive ability exhibit softer wiring distance penalties

**Fig. 5.**
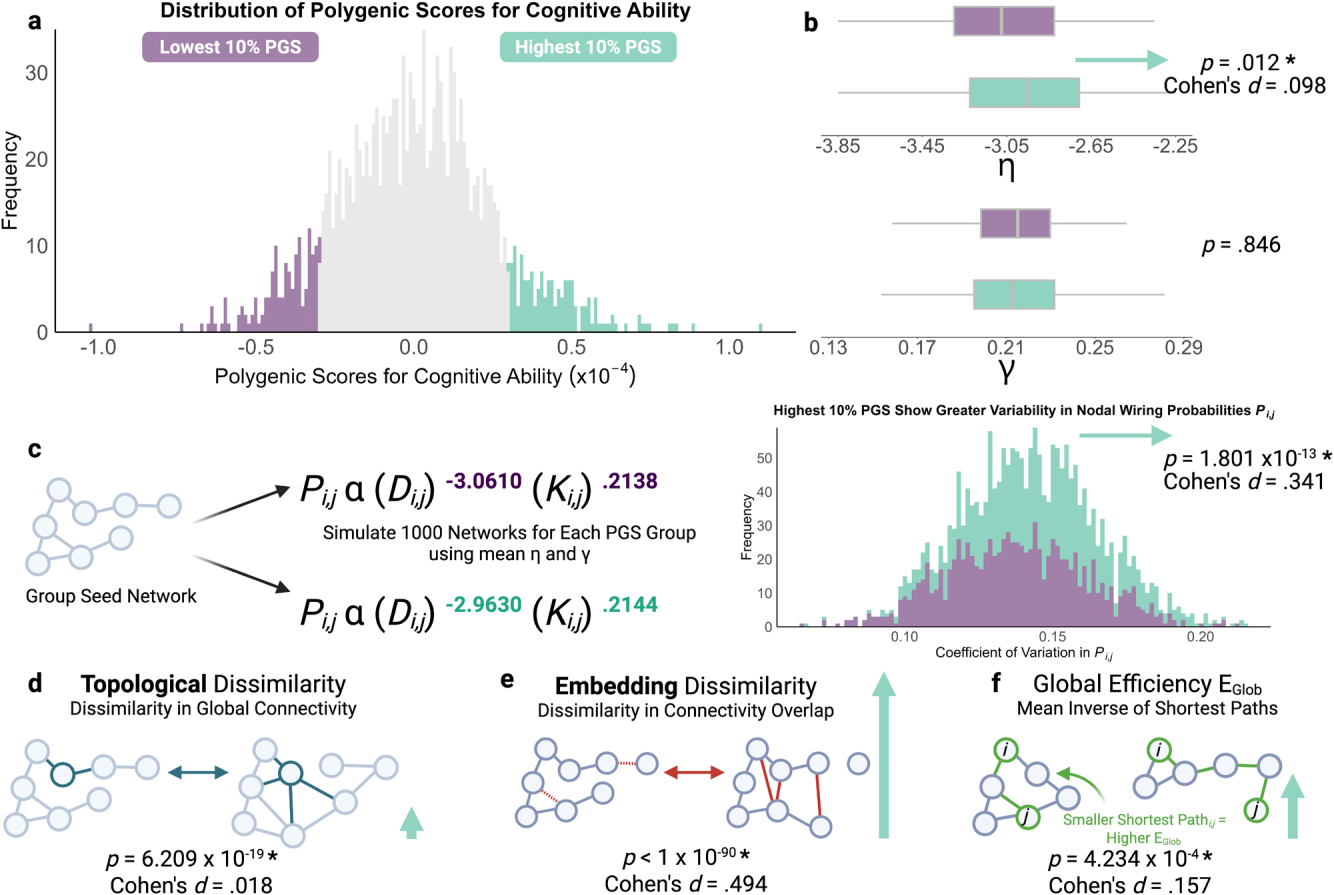
Compared with the bottom 10%, children with the top 10% polygenic scores for cognitive ability display a significantly softer wiring distance parameter η, resulting in more stochastic networks with greater topological dissimilarity, embedding dissimilarity, and global efficiency. (a) We contrasted children with the lowest (purple) and highest (green) 10% polygenic scores for cognitive ability, resulting in 147 children per group. (b) Box plot for mean η and γ for each PGS group. (c) For each PGS group, we simulated 1,000 networks using the neighbours generative rule and group mean η-γ parameters. Children with the largest genetic propensity for cognitive ability displayed a significantly greater frequency of more variable nodal wiring probabilities, with variability quantified as the coefficient of variability. This produces networks significantly higher in three measures. (d) The first is topological dissimilarity, describing differences in distributions of local nodal properties. For example, the node in green has differing topology and length of connections between the two networks, resulting in different topology. (e) The second is embedding dissimilarity, describing differences in the spatial distribution of edges between networks. Differing edges between networks are highlighted in red. (f) The final measure is global efficiency, a proxy of capacity for information transfer in a network. Smaller shortest paths between any two nodes contribute to higher global efficiency, and larger shortest paths to lower global efficiency. Vertical green arrows for (d–f) are proportional to effect sizes.

A simple test of whether there is any relationship between common genetic variants, network organisation, and the economic trade-off at the heart of the GNM is to compare parameters at the extremes. Therefore, we selected participants with the bottom 10% (mean η = -3.061, mean γ = .214), and those with the top 10% (mean η = -2.963, mean γ = .214), genetic propensity for cognitive ability, and compared the parameters for their best-fitting models (Fig. 5a). Group membership (bottom vs. top 10%) had a significant and selective impact on GNM parameters: η differed significantly [*t*(290) = -2.520,*p *= .012, Cohen’s*d*= -.098] between the groups, but γ did not [*t*(290) = -.194,*p *= .846,*d*= -.001]. Put simply, those with the highest genetic propensity had a significantly*softer*distance penalty, relative to their counterparts (Fig. 5b).

Much like emerging brain connectivity, the generative model simulations are probabilistic, and this is an inherent feature of the η-γ trade-off. This means that repeated simulations for each parameter combination will vary subtly. By conducting 1,000 simulations for the optimal parameters for children at the tail ends of the PGS distribution (Fig. 5), we can test directly how this variability differs systematically depending on the parameters of the simulation, such that softer wiring parameters, such as found in the upper end of the PGS distribution, result in increased stochasticity, and this is a potentially adaptive feature of the phenotype of interest. As shown inFigure 5c, taking the average η and γ parameters for each extreme group, and our original seed network, we simulated 1,000 networks per parameter combination with the probabilistic GNM. This provided a direct way of testing for the impact of this difference in wiring parameters on the generative process. The softening of the distance penalty, as seen in those with the highest genetic propensity, does indeed increase stochasticity. The variability in the wiring probabilities across the generative process is significantly higher with the softer η parameter (*p*= 1.801 x 10^-13^,*d*= .341). This raises the possibility that the increased stochasticity observed in networks from children with a higher genetic propensity for cognitive ability is an adaptive phenotype.

There are two ways of considering the diversity of the resulting networks. The first is topological dissimilarity (Fig. 5d): are node degree values similarly distributed across simulations? The second is embedding dissimilarity (Fig. 5e): are connections in the same locations across simulations? The softer distance penalty significantly increases the embedding dissimilarity (*p*< 10^-90^,*d*= .494) but has an extremely small effect of reducing topological dissimilarity (*p*= 6.209 x 10^-19^,*d*= .018). In other words, when the distance penalty is softer, this subtly increases the stochasticity of the generative process, resulting in more structurally diverse outcomes. Finally, does this more stochastic generative process produce more efficient networks? Yes it does: as shown inFigure 5f, global efficiency is subtly but significantly higher when the distance penalty is softer (*p*= 4.234 x 10^-4^, Cohen’s*d*= .157). To examine whether this apparent stochasticity may be an artefact of weaker model fit in one of the groups, we compared their respective energies. The energies in the low PGS (M = .071, SD = .007) and high PGS (M = .072, SD = .007) groups were statistically indistinguishable [*t*(290) = -.075,*p*= .940]. In other words, these parameter differences and resulting simulated network differences do not appear to result from differences in the original model fit.

### 3.6 The distance penalty varies with polygenic scores and network properties

**Fig. 6.**
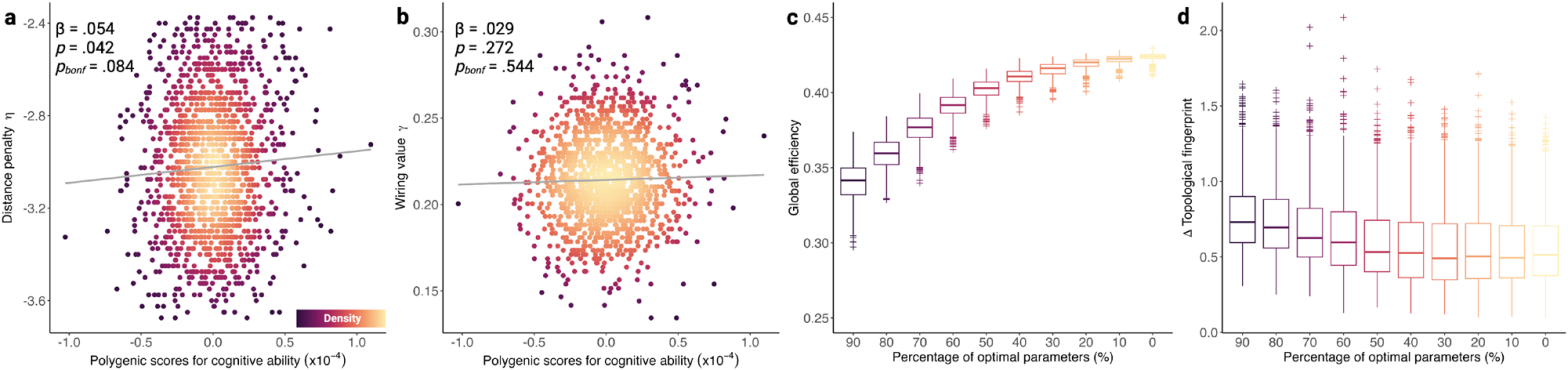
Integrating generative network model parameters, polygenic scores, and network properties. (a) Polygenic scores for cognitive ability are significant positive predictors of the generative distance penalty η, before correcting for multiple comparisons using the Bonferroni (bonf) method. (b) Polygenic scores are not significantly linked to γ. (c) Across 1,000 simulations of deciles of optimal group-level η and γ parameters, an increasingly randomised network topology is linked to increasing global efficiency. (d) The topological dissimilarity between the simulated connectomes in (c) and empirical connectomes rewired using the Maslov–Sneppen algorithm (Maslov & Sneppen, 2002) decreases with softer wiring parameters. Each edge was rewired once, with the degree distribution of the empirical connectomes retained.

Thus far, we have demonstrated significant differences in the distance penalty at the extremes of the PGS distribution. We then turned our attention towards linking GNM parameters with PGSs across all 1,399 children. After controlling for mean framewise displacement, sex, and age, we found that PGSs were significantly associated with a weaker distance penalty η at an uncorrected*p*-value threshold (Fig. 6a, β = .054,*p*= .042*p_bonf_*= .082), but not following a correction for multiple comparisons. PGSs were not significantly associated with γ (Fig. 6b, β = .029,*p*= .272,*p_bonf_*= .544). This extends our 10% decile analysis to the entire sample and provides preliminary evidence that a stronger genetic predisposition to cognitive ability is associated with a weaker penalty against longer connections.

Initially, we planned to compare generative model parameters with empirical network properties, such as global efficiency. However, this is not a fair comparison, for two reasons. First, global efficiency emerges as a trade-off between both parameterised nodal wiring costs and values. This means that, in a simple regression model framework of η predicting empirical global efficiency, even if we control for γ as a covariate, we cannot remove the influence that γ exerted during the simulation process itself. Second, an additional source of variability is the number of connections in the empirical network, which varies naturally across participants. In the empirical connectomes, density is strongly positively correlated with global efficiency [Pearson’s*r*(1397) = .88,*p*= 2.20 x 10^-16^], for instance. This makes it hard to interpret relationships between model parameters and properties of the empirical networks. Therefore, we examined this relationship using two approaches. The first is a simple experiment in which we systematically varied the wiring constraints whilst holding the number of connections constant. In this experiment (Fig. 6c), across 1,000 simulations, we progressively increased network randomness by running the matching-neighbours generative model at 90% (η = -2.620, γ = .220), 80% (η = -2.329, γ = .195), 70% (η = -2.038, γ = .171), 60% (η = -1.747, γ = .146), 50% (η = -1.456, γ = .122), 40% (η = -1.164, γ = .098), 30% (η = -.873, γ = .073), 20% (η = -.582, γ = .049), 10% (η = -.291, γ = .024), and 0% (η = 0, γ = 0) of optimal group-level parameters (η = -2.911, γ = .244), using the group-level seed and group-level target. As η and γ approach 0, indicating increasing network randomness, global efficiency increases. Further, topological dissimilarity between simulated networks to 1,000 randomly rewired empirical connectomes decreased with looser wiring constraints, confirming a more randomised topology (Fig. 6d). In essence, as you loosen the wiring parameters you add more randomness and create more efficient networks. The second way of looking for relationships between wiring parameters, randomness, and global efficiency is by simulating with parameters belonging to participants at the tail ends of the polygenic score distribution, where the number of connections is held constant. As previously demonstrated (Fig. 5b), children with stronger polygenic scores for cognitive ability had significantly looser wiring constraints, resulting in more globally efficient simulated networks.

### 3.7 Overlapping gene ontologies for cognitive ability polygenic scores and generative structural brain organisation parameters

**Fig. 7.**
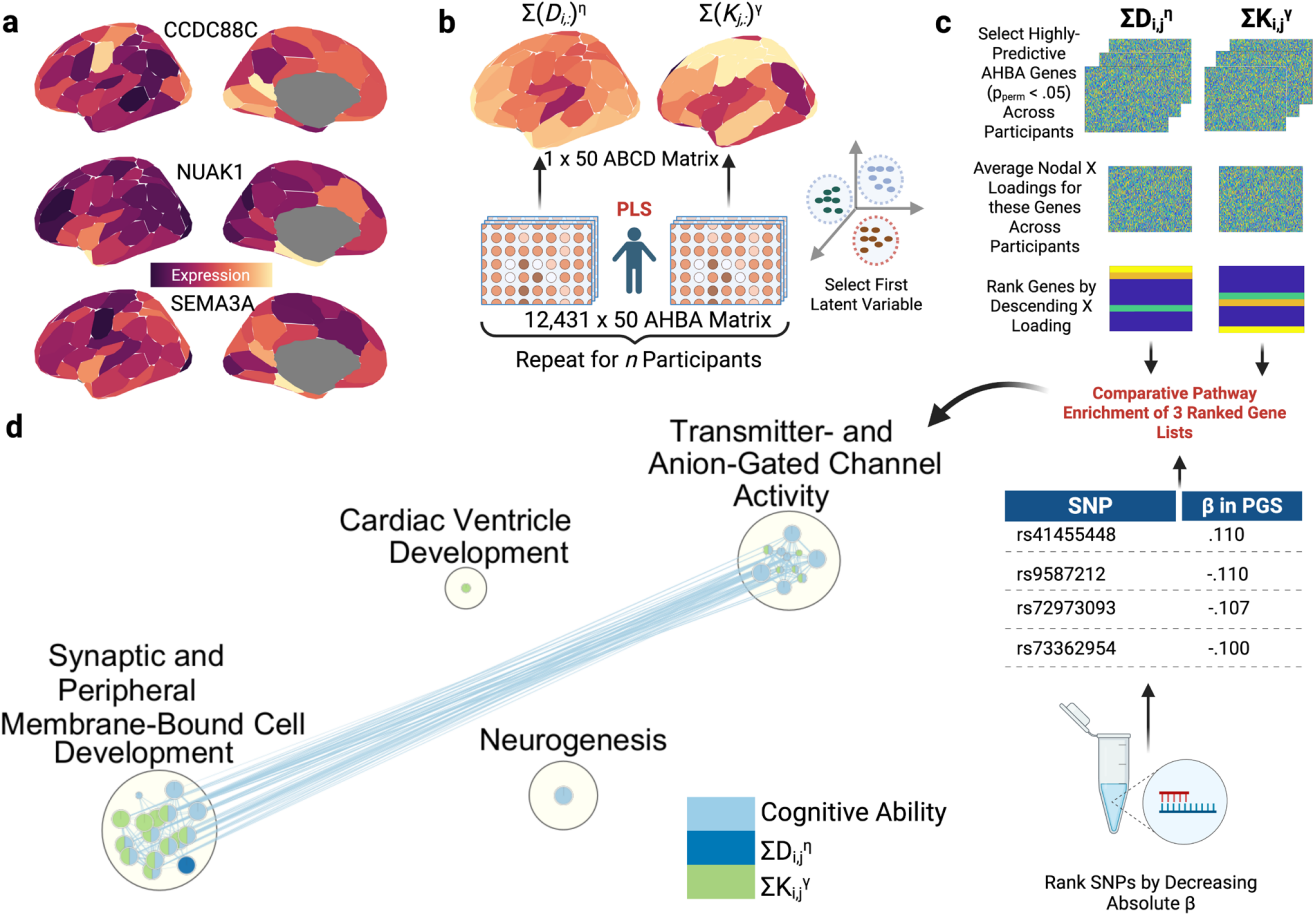
Triangulating the genomic basis of parameterised nodal η, γ, and cognitive ability PGSs. (a) The spatial distribution of genes previously highlighted by the literature as being predictive of structural connectivity (Wainberg et al., 2024). (b) For each participant, two PLSs are conducted, using RNA-sequencing genomic data from the left hemisphere of the Allen Human Brain Atlas (AHBA) as predictors of parameterised nodal wiring costs or values, respectively, constrained to the left hemisphere. In total, 10,000 permutations generate a null distribution for each gene’s ability to predict parameterised nodal wiring costs and values, respectively. (c) For each parameter, each matrix has dimensions 2,153 (number of participants) x 12,431 (number of genes). Three matrices are presented for each parameter, each representing a PLS component. Using the first component for each parameter, representing the principal axis of genetic variation linked to parameterised nodal wiring costs or values, we identified genes with a permuted*p*-value (*p_corr_*) of less than .05 for at least one participant. We then averaged PLS loadings across all participants for the first component for each parameter, yielding two 12,431 x 1 matrices, and extracted the loadings associated with genes with*p_corr_*< .05. The resultant list of genes associated with parameterised nodal wiring costs or values across at least one participant were then ranked by descending PLS loading and submitted to an ordered pathway enrichment analysis. Short nucleotide polymorphisms (SNPs) from the cognitive ability polygenic scores are ranked by decreasing contributions (absolute β). (d) To compare the genomic bases of the three lists, comparative gene ontology analysis is performed using g:Profiler, significant at*p*< .01. Parameterised nodal wiring value and cognitive ability converge on gene ontologies describing synaptic, peripheral, and membrane-bound cell development. Parameterised nodal wiring costs also share the same cluster, but not nodes, and, therefore, make an independent contribution. Cognitive ability PGSs only converge with parameterised nodal wiring value, the greatest converge of which is in cell development. The size of each node represents the number of genes in that ontology. Each node represents a distinct pathway, where node size is proportional to gene set size. The blue lines (edges) connecting clusters represent the degree of overlap of genes between distinct pathways, determined using the gene set similarity coefficient. In our case, we used an edge cutoff threshold of .5, where .375 represents the densest network and 1 represents the sparsest. Overlap will be greater within clusters than between clusters. An example of strong inter-cluster connectivity is the relationship between GO:0030594, representing neurotransmitter activity, and GO:0007268, representing chemical synaptic transmission, with a similarity coefficient of .54. The weakest inter-cluster relationship and, therefore, smallest overlap is between GO:0098916, representing anterograde trans-synaptic signalling, and GO:0098793, representing the pre-synapse, with a similarity coefficient of .38.

Finally, we tested whether the genomic correlates of η, γ, and PGSs to converge on common biological aetiologies. If they do, this may shed light onto which biological ontologies are particularly important in bridging common genomic variation linked to cognition and the cost–value trade-off needed to simulate connectomes. To investigate this, we first examined genomic predictors of parameterised nodal wiring costs. For each participant, we conducted two PLSs using microarray (validated by RNA-sequencing) regional gene expression matrices (seeFig. 7a) from the Allen Human Brain Atlas (AHBA), with parameterised nodal wiring costs or value, respectively, as the response variable (Fig. 7b). We extracted AHBA genes which significantly (*p_perm_*< .05) predicted either parameterised nodal wiring costs or value across all participants, averaged their nodal loadings onto the first PLS latent variable across participants, and ordered the genes by descending loading (Fig. 7c). To create a gene list for the PGS, we used all 76,745 SNPs in the PGS, ranked by descending absolute β.

To assess possible shared ontologies of η, γ, and PGSs, we submitted all three ranked gene lists to a multi-query comparative pathway enrichment analysis. We refer the reader to theSupplementary Methodsfor details on the genetic enrichments of η, γ, and PGSs. Pathway enrichment analysis tests whether inputted genes are significantly more likely to be grouped together compared with chance. In the case of ordered gene lists, the algorithm searches for the largest sub-list of genes significantly associated with an ontology and adjusts for multiple comparisons of interdependent gene ontologies (Raudvere et al., 2019;Reimand et al., 2019). This produced three adjusted*p*-values for each pathway, which represents the likelihood that each gene list was significantly enriched compared with chance (seeSupplementary Table S7for each pathway’s top enriched categories). Ninety-six gene ontologies were significantly enriched for at least one predictor (*p_adj_*< .01), and mostly for molecular functions (43.75%). Whilst 951 AHBA genes were significantly linked to nodal wiring costs and 561 for nodal wiring value, we observed greater overlap in enriched ontologies between nodal wiring value and the cognition PGS (20 ontologies enriched, adjusted*p*< .05), than between nodal wiring cost and the cognition PGS (9 ontologies enriched, adjusted*p*< .05). Therefore, whilst nodal wiring cost was associated with stronger cortex-restricted donor-derived genetic variation overall, the genetic variation for nodal wiring value overlapped to a*greater extent*with the cognition PGS.

When grouped on connectivity strength, four ontological groups emerged (Fig. 7d), two of which were shared by all predictors. Greater convergence between parameterised nodal wiring value and cognitive ability PGSs occurred for synaptic, peripheral, and membrane-bound cell development, and less for transmitter and anion-gated channel activity. Whilst parameterised nodal wiring cost also contributed to synaptic, peripheral, and membrane-bound cell development, it made a unique contribution towards presynaptic development. Cognitive ability PGS genes were distinguished by neurogenesis, and parameterised nodal wiring value by cardiac ventricle development. Individual gene ontologies are accessible at the project’s GitHub repository.

## 4 Discussion

Human brain development proceeds via dynamic interactions between our unique genetic background, our experience, and physical constraints on the formation of complex neuronal networks (Gottlieb, 2007;Kaiser & Hilgetag, 2004;Westermann et al., 2007). One popular way of conceptualising those physical constraints is as an economic trade-off between the metabolic cost of creating or maintaining new connections and the topological value those connections provide (Akarca et al., 2021,^2022^;Betzel et al., 2016;Bullmore & Sporns, 2012;Carozza et al., 2022;Vértes et al., 2012;Zhang et al., 2021). In this framework, structural connectivity can be conceptualised as a negotiated trade-off, maximising topological value whilst minimising cost. Generative modelling, whilst not mechanistic per se, provides us with a way of capturing that trade-off by testing whether it can recapitulate the high-level organisational features of the observed networks. We investigated whether and how population-level variability in overall brain organisation, and the trade-off you need to simulate it, are related to known genetic variants associated with cognitive performance (Savage et al., 2018). Across 2,154 children, a homophily based model provided the best account of structural brain organisation. In other words, accurate simulations were those in which topologically valuable connections were those that formed between nodes with similar connectivity profiles. The cost–value trade-off needed to simulate structural brain organisation differs according to someone’s polygenic propensity for cognitive ability. At the extremes, this is most apparent as a softening of the distance penalty, which in the simulation gives rise to more stochastic, diverse, and efficient networks for those with the highest PGS. A comparative pathway enrichment analysis shows that these known variants converged on common genetic ontologies with wiring costs η and value γ. Put simply, population-level genetic variability that is linked to cognitive performance overlaps with variability in generative wiring parameters. One possible reason is because of shared cellular and molecular mechanisms, with regional co-expression with topologically valuable areas.

We take two approaches to integrate the modelling with genetics, and they give apparently different outcomes. But these two approaches are asking different questions, are sensitive to different kinds of individual differences, and, therefore, inevitably reveal different relationships. The first approach was to compare parameters across individuals, ordered by the PGS. This approach is sensitive to the*magnitude*of those parameters, but not their*regional distributions*. Since η varies most from individual to individual, relative to γ, this is most likely to identify relationships with η. Crucially, however, the η does not vary regionally across individuals, because this is calculated from the parcellation coordinates which are identical across the sample. The second approach was to conduct a comparative gene enrichment for wiring parameters and PGSs across the entire sample to establish the points of intersection between these predictors. The second approach—using donor-derived expression values to find relationships with the genes within the PGS restricted to those expressed in the brain—is sensitive to the*distribution*of model parameters across the cortex, but not individual differences in their magnitude. From a biological standpoint, wiring cost is determined by distance, with set metabolic costs, and is physically constrained by a parcellation, thus varies minimally between participants. By contrast, the distribution of γ varies markedly, as it updates dynamically. Thus, the PLS approach is much more likely to identify genes meaningfully associated with individual differences in γ. Together, we demonstrate that that each part of the wiring equation significantly relates to different sources of genetic variation. That is, at a behavioural level, η was significantly associated with the cognition PGS. However, at a genomic level, genomic enrichments of the cognitive ability PGS overlapped most to γ, whilst the AHBA genetic signal overall was stronger for η. This overarching association of stochasticity with wiring value occurs*in tandem*with associations between stochasticity and wiring cost in a subset of participants at the extreme tails of a genotypic, namely PGS, distribution.

In line with prior work across species, scales, and neurodevelopmental conditions (Akarca et al., 2021,^2022^;Betzel et al., 2016;Carozza et al., 2022;Zhang et al., 2021), we found a generative model combining wiring cost and homophilic value could most accurately simulate the high-level properties of observed structural brain networks. This suggests that prioritising forming edges between nodes with shared neighbours is a major organisational principle for formation of real networks. This reflects a key organisational principle for the economy of emerging structural connectivity, whereby topological neighbours are also likely to be anatomical neighbours (seeBullmore & Sporns, 2012). Interestingly, our finding also aligns with network neuroscience accounts of general cognitive ability (Barbey, 2018) emerging from a trade-off between minimising wiring cost, which prefers local efficiency, with maximising wiring topology, which prefers global efficiency.

Having a high genetic propensity for cognitive ability was significantly associated with a weaker distance penalty, resulting in more stochastic, diverse, and globally efficient networks. Stochasticity in neural networks, particularly in terms of wiring, emerges across multiple scales, ranging from genetic splicing, epigenetic chromatin modifications, and differing number of cell adhesion and intra-cellular signalling molecules steering neuronal growth cones to build a connectome (Honegger & de Bivort, 2018). This stochasticity may be evolutionarily adaptive in several ways (Honegger & de Bivort, 2018), such that sufficient variation in the population allows members to display adaptive behaviours which are either stable (diversified bet-hedging) or vary (phenotypic plasticity), and this requires fewer successive generations to achieve (gene saving) compared with reduced stochasticity. We show, in our simulations at least, that common genomic variation in the population associated with cognitive ability encodes greater stochasticity, resulting in more efficient networks. Notably, through repeated simulations of generative parameters belonging to the tail ends of the distribution of polygenic scores, we systematically show that greater stochasticity is an inherent feature of the networks and may provide an adaptive advantage, in line with prior work showing that the generative trade-off can be shifted when organisms develop in unpredictable circumstances (Carozza et al., 2023). Our findings show that relatively small, albeit significant, changes in the wiring parameters can have a disproportionate impact on network stochasticity and diversity, presumably because the process of network formation is probabilistic, so even small shifts in economic constraints will cascade across the generative process. However, one caveat of our findings is that whilst the model fit of simulated networks between the high and low PGS groups was statistically indistinguishable, we cannot conclude that the empirical networks themselves formed with greater stochasticity. One potentially fruitful way forward could be to combine this modelling with cellular recordings over days, not years, to directly match stochasticity at a cellular level with modelling of this kind (Akarca et al., 2022).

The specificity of the relationship between genes, emerging structural connectivity, and cognitive ability depends on variability in genetic relatedness and the influence of individual genes. At the extreme of genetic relatedness, twin studies have demonstrated genetic influences on specific aspects of network topology, such as rich-club nodes with weak peripheral edges, as well as inter-hub connectivity (Arnatkeviciute et al., 2021). At the extreme of genetic influence, high-impact single-gene mutations, implicated in neurogenetic disorders, exhibit tight spatial coupling with altered structural brain organisation. Transcriptional vulnerability suggests that differences in regional gene expression profiles shape ongoing structural connectivity, with knock-on implications for cognition. A prior study validated this model in participants with neurogenetic conditions, whereby patterns of morphometric dissimilarity were most strongly associated with copy number variation, and the spatial patterns could be recovered in gene expression maps derived from control post-mortems (Seidlitz et al., 2020). Our findings reflect the opposite end of the spectrum—common genetic variation in non-related neurotypical children. Thus, inevitably the overall strength of the genetic influences is smaller because they reflect population-level variation, rather than that between twins or following a high-penetrance mutation.

The present study also contributes to a wider debate about the utility of the insights provided by generative modelling of connectomes alongside more standard graph theory metrics. We argue that whilst graph theory provides a simple summary of connectome properties, these are descriptive. Generative models provide additional insight because they quantify whether and how high-level topological properties of networks might arise from simple underlying changes in constraints—in this example, from the distance penalty. The specificity of the relationship between each part of the generative wiring equation with either participant-derived genome-wide or donor-derived cortex-wide genetic variation highlights the additional insights afforded by the generative modelling approach.

Whilst the current study was the first to triangulate cognition, participant-derived and donor-derived genetics with structural connectivity metrics at a population level, there are inevitable limitations. First, our sample is slightly overrepresented for children with parents with higher educational backgrounds and of European and Asian descent. Second, we included participants with high-quality neuroimaging data which could skew our sample towards those with low levels of hyperactivity and associated head motion. Third, we established genomic patterning of GNM parameters using adult post-mortem atlases. Whilst recent efforts have been made to collate post-mortem gene expression data across the lifespan (Jiao et al., 2019), including childhood, these offer considerably less spatial resolution than the AHBA (see work byArnatkevic̆iūtė et al., 2019for further discussion of AHBA limitations). Fourth, previous work has suggested alternative generative modelling specifications, which may challenge the conclusion that homophily based models perform best. For example, a variation accounting for change in topology as part of a growth model outperformed static models (Oldham et al., 2022), such as those used in the current study. Further, explicitly incorporating genetic constraints improved model performance, which otherwise fails to recapitulate spatial topography in absence of a seed network (Arnatkeviciute et al., 2021;Oldham et al., 2022). Finally, the current GNM specification is binary, with a single pair of parameters describing global development, adding a single edge at each iteration, and assumes that the morphological and microstructural properties of these connections are consistent across development and the cortex. Inevitably, synaptic connections are not added in a binary fashion but are gradually strengthened or weakened through experience-dependent synaptic plasticity, and in other cases completely pruned (Huttenlocher & Dabholkar, 1997). Advances in the complexity of generative models will be needed to capture this nuance. The current implementation of GNMs is designed to capture the high-level topological properties of connectomes, not the formation of specific connections.

In summary, trade-offs between nodal wiring cost and value accurately recapitulated the emergence of structural connectivity properties of 2,153 children. Focusing on a subset of children at the extreme tails of a distribution for polygenic scores revealed a weaker distance penalty, which has a disproportionate impact on network stochasticity, diversity, and efficiency in the resulting simulations. Whilst polygenic scores for cognitive ability were associated with weaker distance penalties, the latter of which had a stronger AHBA enrichment than wiring value, AHBA cortex-restricted genes overlapped most with wiring value constraints. Together, we demonstrate how shared underlying cellular and biological processes may shape variability in structural connectivity and cognitive ability in childhood.

## Supplementary Material

Supplementary Material

## Data Availability

All analysis scripts and gene ontologies, including detailed documentation, are provided athttps://github.com/AlicjaMonaghan/abcd_genes_brain_cognition. Structural connectomes were generated using QSIprep 0.15.3 (Cieslak et al., 2021). All generative modelling was conducted in MATLAB R2018. Additional analyses were conducted in R version 4.2 and Python version 3.8. We used data from Release 4 of the ABCD study (http://dx.doi.org/10.15154/1523041). Figures were generated using BioRender.
