# Supplementary material for: Brain wiring economics, network organisation and population-level genomics
Source: Imaging Neurosci (Camb). 2025 Jun 4;3:IMAG.a.31. doi: 10.1162/IMAG.a.31 (PMC12319759; doi:10.1162/IMAG.a.31)
Supplement: Supplementary Material [file imag.a.31_supp.pdf]

Brain wiring economics vary with population-level genomics

Alicja Monaghan[1,2]*, Danyal Akarca[1] and Duncan E. Astle[1,3]

Supplementary material

Table 1. Descriptive statistics for the distributions of local and global graph theory measures across 3 parcellations of varying granularity. All are local measures, except density, and are defined in the Results.

	Parcellation					
	Schaefer 100-Node		Brainnetome 246-Node		Schaefer 400-Node	
Metric	Mean (SD)	Range	Mean (SD)	Range	Mean (SD)	Range
Density	6.55% (.48)	2.24 - 7.35	2.04% (.19)	.34 - 2.35	.49% (.05)	.03 - .59
Degree	6.49 (3.17)	.65 - 15.57	4.99 (3.38)	0 - 19.91	1.95 (2.08)	0 - 11.30
Clustering	.42 (.18)	0 - .89	.34 (.20)	0 - .78	.15 (.18)	0 - .76
Betweenness Centrality	239.44 (290.42)	0 - 1583.39	824.24 (996.12)	0 - 6494.36	390.59 (812.89)	0 -6910.37
Eigenvector Centrality	.07 (.07)	0 - .24	.03 (.05)	0 - .31	.01 (.04)	0 - .23
Local Efficiency	.57 (.21)	0 - .91	.43 (.24)	0 - .84	.17 (.22)	0 - .82
Mean Total Edge Length	246.10 (177.23)	11.42 - 1077.48	163.01 (140.50)	0 - 736.61	44.84 (50.97)	0 - 257.05

Table 2. Polygenic score model specification for European and Non-European subsets. For each model, 6 principal components (PC) of ancestry were included as covariates. *Note.* * and ** represent significance at $p < .01$ and $p < .001$, respectively. Clumping thresholds of $p = .1$ and $p = .2$ were used for European and Non-European participants, respectively.

Ancestry	*Parameter*	*Coefficient*	*Standard Error*	*t-value*	*Pr(>|t|)*
European	Intercept	.456	$2.924e^{-2}$	15.589	$1.307e^{-53}$ **
	g Factor Loading	26234.927	1824.743	14.377	$5.196e^{-46}$ **
	Sex (Male)	$-1.011e^{-1}$	$4.029e^{-2}$	-2.510	.012 *
	PC1	3.008	1.526	1.971	.049
	PC2	-14.120	1.470	-9.601	$1.183e^{-21}$ **
	PC3	5.241	1.600	3.276	.001 **
	PC4	-1.685	1.469	-1.146	.252
	PC5	-3.393	1.472	-2.305	.021
	PC6	-6.737	1.476	-4.564	$5.130e^{-6}$ **
Non-European	Intercept	-.372	$6.167e^{-2}$	-6.026	$2.235e^{-9}$ **
	g Factor Loading	18998.573	4164.188	4.564	$5.578e^{-6}$ **
	Sex (Male)	-.121	.008	-1.432	.152
	PC1	.980	1.505	.652	.515
	PC2	4.892	1.494	3.274	.001 **
	PC3	-7.633	1.454	-5.249	$1.810e^{-7}$ **
	PC4	-2.006	1.461	-1.373	.170
	PC5	-2.519	1.475	-1.708	.088
	PC6	-.539	1.454	-.371	.711

Table 3. Polygenic model performance for varying clumping thresholds across European and non-European subsets.

Ancestry	*Polygenic Clumping Threshold P_T*	*Polygenic Model Fit R^2*	*p*	*β*	*Standard Error*
European	.001	.025	$3.359e^{-32}$	2225.124	187.124
	.05	.038	$1.969e^{-48}$	9579.576	648.438
	.1	.040	$5.070e^{-51}$	13024.307	857.635
	.2	.039	$7.729e^{-50}$	17479.276	1165.373
	.3	.038	$6.215e^{-48}$	20755.122	1412.636
	.4	.037	$2.958e^{-47}$	23757.929	1629.252
	.5	.036	$5.196e^{-46}$	26234.927	1824.743
Non-European	.001	.012	$1.053e^{-4}$	1637.998	420.961
	.05	.012	$1.188e^{-4}$	5410.462	1401.212
	.1	.014	$3.557e^{-5}$	7804.361	1880.474
	.2	.017	$2.867e^{-6}$	12194.429	2593.133
	.3	.017	$4.226e^{-6}$	14646.042	3169.217
	.4	.016	$9.407e^{-6}$	16434.281	3693.490
	.5	.016	$5.578e^{-6}$	18998.574	4164.188

Table 4. Group-level model energy across simulation thresholds. Note. “Sptl” = Spatial, “Neighbours” = Number of shared neighbours, “Matching” = Matching index, “Clu-Avg” = Average clustering, “Deg-avg” = Average degree.

	Generative model energy across simulation thresholds M (SD)				
Model	Top	Top 10	Top 25	Top 500	Top 1000
sptl	.205	.209 (.002)	.212 (.004)	.230 (.007)	.236 (.008)
neighbours	.090	.108 (.010)	.121 (.013)	.197 (.032)	.241 (.054)
matching	.100	.107 (.005)	.118 (.011)	.187 (.030)	.223 (.044)
clu-avg	.120	.126 (.005)	.128 (.003)	.146 (.007)	.154 (.010)
deg-avg	.148	.150 (.001)	.156 (.005)	.186 (.014)	.202 (.020)

Table 5. Lowest-energy group simulations with associated optimal η and γ parameters, across 3 parcellations. Each generative rule was evaluated for 99,856 unique combinations of η [-7 ≤ η ≤ 7] and γ [-7 ≤ γ ≤ 7]. *Note.* “Clu-Avg” = Average Clustering Coefficient, “Deg-Avg” = Average Degree.

	Parcellation								
	Schaefer 100-Node			Brainnetome 246-Node			Schaefer 400-Node		
Rule	Energy	η	γ	Energy	η	γ	Energy	η	γ
Clu-Avg	.120	-6.422	-4.822	.228	-4.200	-2.289	.173	-5.089	1.000
Deg-Avg	.148	-5.578	1.667	.240	-4.556	1.978	.138	-3.533	1.444
Matching	.100	-3.800	.333	.106	-2.467	.333	.110	-2.956	.467
Neighbours	.090	-2.911	.244	.110	-2.200	.289	.107	-2.911	.378
Spatial	.205	-6.378	4.289	.289	-4.111	.244	.268	-3.578	3.400

Table 6. Mean topological dissimilarity (TD) and correlations between simulated and observed degree for 1000 simulations of each generative model’s lowest energy η and γ combination, across 3 parcellations. For the Brainnetome 246-node parcellation, the models differed significantly in their topological dissimilarity [$F(4,4999) = 1654.00$, $p < .001$] and ability to capture observed degree [$F(4,4999) = 1643.50$, $p < .001$], with all post-hoc comparisons highly significant ($p < .001$). For the Schaefer 400-node parcellation, the models also differed significantly in their topological dissimilarity [$F(4,4999) = 4502.14$, $p < .001$], with all post-hoc comparisons highly significant ($p < .001$). The models also differed in their correlation with observed degree [$F(4,4999) = 2197.93$, $p < .001$], with all post-hoc comparisons highly significant, apart from the two homophily models performing similarly to each other ($p = .991$). *Note.* “Clu-Avg” = Average Clustering Coefficient, “Deg-Avg” = Average Degree.

	Schaefer 100-Node	
GNM Rule	Topological Dissimilarity (Mean ± SD)	Simulated – Observed Degree Pearson's *r* (Mean ± SD)
Clu-Avg	1.975 ± .156	.165 ± .071
Deg-Avg	.963 ± .236	.343 ± .062
Matching	1.004 ± .280	.394 ± .079
Neighbours	.979 ± .265	.412 ± .077
Spatial	1.058 ± .339	.130 ± .062
	Brainnetome 246-Node	
Clu-Avg	1.683 ± .132	.050 ± .062
Deg-Avg	1.462 ± .264	.076 ± .031
Matching	1.035 ± .239	.119 ± .046
Neighbours	1.098 ± .252	.129 ± .050
Spatial	.973 ± .281	-.018 ± .039
	Schaefer 400-Node	
Clu-Avg	1.482 ± .188	-.095 ± .120
Deg-Avg	.998 ± .160	.197 ± .044
Matching	.618 ± .187	.016 ± .070
Neighbours	.577 ± .194	.015 ± .073
Spatial	.763 ± .138	-.055 ± .044

Summary of Gene Ontologies for Parameterised Nodal Wiring Costs, Value, and Cognitive Ability Allen Human Brain Atlas (AHBA) genes predicted parameterised nodal wiring costs and values, separately, for each participant, through partial least squares regression. Following 10,000 permutations for each participant, AHBA genes with permuted *p*-values less than .05 across all participants were selected and then ranked by decreasing mean loading onto the first latent variable. 76,745 short-nucleotide polymorphisms were ranked by decreasing absolute β in the cognitive ability polygenic score. All gene lists were submitted separately to g:Profiler (Kolberg et al., 2020) for gene enrichment, with a cut-off of $p < .05$ corrected for multiple comparisons, and default parameters. Electronic annotations were excluded for robustness.

To examine the functional roles of these genes, we performed ordered pathway enrichment analysis for each list separately, using g:Profiler (Kolberg et al., 2020; Raudvere et al., 2019; Reimand et al., 2019). Pathway enrichment analysis tests whether inputted genes are significantly more likely to be grouped together compared to chance. In the case of ordered gene lists, the algorithm searches for the largest sub-list of genes significantly associated with an ontology, and adjusts for multiple comparisons of interdependent gene ontologies (Raudvere et al., 2019; Reimand et al., 2019).

Parameterised nodal wiring costs were significantly enriched for 19 pathways, most of which linked to molecular functions (73.68%). The strongest enrichments were for genes associated with transmembrane transporter activity of metal ions ($p_{adj} = 2.116 \times 10^{-4}$), cations ($p_{adj} = 8.192 \times 10^{-4}$), and inorganic molecules ($p_{adj} = .004$). Parameterised nodal wiring value were significantly enriched for 41 pathways, most of which linked to biological processes (43.90%). Rather than transporter activity, the strongest enrichments were for genes associated with different modes of signalling, including synaptic ($p_{adj} = 1.130 \times 10^{-5}$), cell-cell ($p_{adj} = 1.949 \times 10^{-5}$), and anterograde trans-synaptic ($p_{adj} = 2.348 \times 10^{-5}$).

Next, we submitted all 76,745 SNPs in the PGS, ranked by descending absolute β, to pathway enrichment analysis. SNPs predictive of cognitive ability were significantly enriched for 297 pathways, mostly encoding biological processes (49.50%). The strongest enrichments were for genes associated with synaptic ($p_{adj} = 3.485 \times 10^{-29}$) and post-synaptic ($p_{adj} = 3.684 \times 10^{-26}$) membranes, alongside synaptic ($p_{adj} = 7.602 \times 10^{-20}$) and trans-synaptic ($p_{adj} = 3.821 \times 10^{-20}$) signalling. Summaries for all pathway enrichment analyses and g:Profiler links are provided below.

Table 7. Top enriched gene ontology categories for polygenic scores for cognitive ability, parameterised nodal wiring costs, and parameterised nodal wiring value. *Note*. "MF" = Molecular function; "BP" = Biological processes; "CC" = Cellular Components.

	Parameterised Nodal Wiring Costs	*Parameterised Nodal Wiring Value*	*Cognitive Ability*
Number of Genes	951	561	15,234
g:Profiler Link	https://biit.cs.ut.ee/gplink/l/m2E_uLK0TG	https://biit.cs.ut.ee/gplink/l/T8OtQThDQ_	https://biit.cs.ut.ee/gplink/l/lGxCWYZpSo
% BP/CC/MF	10.526% / 15.790% / 73.684%	43.902% / 29.268% / 26.829%	49.495% / 27.609% / 22.896%
Top 10 Enriched Categories	GO:0046873, Metal Ion Transmembrane Transporter Activity (p_{adj} = 2.116e^{-4})	GO:0099536, Synaptic Signalling (p_{adj} = 1.130e^{-5})	GO:0097060, Synaptic Membrane (p_{adj} = 3.485e^{-29})
	GO:0005215, Transporter Activity (p_{adj} = 4.480e^{-4})	GO:0007267, Cell-Cell signalling (p_{adj} = 1.949e^{-5})	GO:0045202, Synapse (p_{adj} = 2.586e^{-26})
	GO:0008324, Cation Transmembrane Transporter Activity (p_{adj} = 8.192e^{-4})	GO:0098916, Anterograde Trans-Synaptic signalling (p_{adj} = 2.348e^{-5})	GO:0098794, post-Synapse (p_{adj} = 3.684e^{-26})
	GO:0022890, Inorganic Cation Transmembrane Transporter Activity (p_{adj} = 9.618e^{-4})	GO:0007268, Chemical Synaptic Transmission (p_{adj} = 2.348e^{-5})	GO:0045211, Post-Synapse Membrane (p_{adj} = 2.072e^{-24})

AHBA Pre-Processing

In brief, probes were reannotated with up-to-date genetic labels from Arnatkevičiūtė and colleagues (2019), and intensity-based filtering performed, such that only probes whose expression exceeded background noise in at least 50% of brain regions were retained. In instances where multiple probes mapped onto the same gene, the probe with the highest correlation in RNA-sequencing data between the two donors with such data were selected. Note that whilst different probe selection approaches exist, RNA-sequencing arguably offers the highest validity, due to reduced noise and lack of reliance on known genetic associations (Arnatkevičiūtė et al., 2019). However, since only two donors have such data, we decided to use RNA-sequencing for probe selection and as an external reference for the microarray data, for which all 6 donors have data. The MNI coordinates of the AHBA samples were then updated using the *alleninf* Python package (Gorgolewski et al., 2014), and mapped to each voxel in the Schaefer 100-node parcellation with a distance threshold of 2mm. Regional gene expression values for each donor were then normalized using a scaled robust sigmoid function previously shown to be robust to outliers (Arnatkevičiūtė et al., 2019; Fulcher & Fornito, 2016). To ensure consistency of gene expression values with RNA-sequencing, we excluded microarray genes absent in the RNA-sequencing matrices and whose Spearman's correlation with RNA-sequencing gene expression exceeded .75. We then averaged across donors to produce a 100 (number of nodes) x 12431 (number of genes) matrix. We restricted our analyses to the left hemisphere, for which all 6 donors had data, yielding a dense 50 x 12431 matrix.

Graph theory measures

Degree

k_i is the number of connections node *i* has (Rubinov & Sporns, 2010):

$$k_i = \sum_{j \in N} a_{ij}$$

Clustering coefficient

We computed the Watts-Strogatz clustering coefficient *C* (Watts & Strogatz, 1998) for unweighted, undirected binary networks at each node *i*. t_i is the number of triangles node *i* is part of, where a triangle is a set of three nodes connected by three vertices and edges, whilst k_i is the number of possible neighbours node *i* can have. Therefore, a node with a high clustering coefficient will be part of many possible triangles:

$$C_i = \frac{2t_i}{k_i\,(k_i - 1)}$$

The global clustering coefficient was simply the clustering coefficient averaged across all nodes:

$$C = \frac{1}{n}\sum_{i \in N} C_i$$

Betweenness centrality

Betweenness centrality measures the proportion of shortest paths a given node *i* passes through (M. E. J. Newman, 2005). *n* is the total number of vertices in the network, $g_i^{(st)}$ are the number of geodesic paths between vertices *s* and *t* which pass through node *i*, and n_{st} are the total number of geodesic paths between *s* and *t*:

$$b_i = \frac{\sum_{s<t} g_i^{(st)}/n_{st}}{\frac{1}{2}n(n-1)}$$

Modularity

Modularity *Q* measures the extent to which nodes in a network can be divided into non-overlapping communities which maximise within-community edges and minimise between-community edges (M. E. J. Newman, 2006; M. E. J. Newman & Girvan, 2004). This is quantified by comparing community structure in empirical networks to that of random networks. The Brain Connectivity Toolbox (2009) calculates modularity based on a formulation from Newman and Girvan (2004). In a symmetric *k* x *k* matrix *e*, e_{ii} is the fraction of all edges in the network that connect to vertices within community *i*, representing intra-modular connectivity. a_i is equal to $\Sigma_j e_{ij}$, which is the fraction of vertices in community *j* linking with community *i*, summed across all vertices in *j*, representing inter-modular connectivity. Modularity range between 0, representing no community structure beyond what would be expected at random, to 1, representing strong community structure:

$$Q = \sum_i (e_{ii} - a_i^2) = Tr\,\boldsymbol{e} - \| \boldsymbol{e}^2 \|$$

Eigenvector centrality

Whilst, the degree of each node is the sum of its connections to all other nodes, assuming such connections are equal, eigenvector centrality measures the tendency of each node to connect with other high-centrality nodes (M. E. Newman, 2008). To calculate nodal eigenvector centrality, compute the eigenvector *V* and corresponding eigenvalues of the connectivity matrix, find the index of the largest eigenvalue, and extract the corresponding column of *V*.

Participation coefficient

The participation coefficient of node *i* (P_i) measures the distribution of its connections amongst all modules. A high participation coefficient suggests that connections are uniformly distributed across systems, whereas a low participation coefficient suggests that connections are restricted to a single system (Guimerà & Nunes Amaral, 2005):

$$P_i = 1 - \sum_{s=1}^{N_M} \left(\frac{k_{is}}{k_i}\right)^2$$

N_M is the number of modules, k_i is the total number of connections *i* has, and k_{is} is the number of connections node *i* has in module s.

Efficiency

We calculated global efficiency *E* according to Latora and Marchiori (2001) where *n* is the number of nodes, *N* is the set of all nodes, E_i is the efficiency of node *i*, and d_{ij} is the shortest path length between nodes *i* and *j* (Rubinov & Sporns, 2010):

$$E = \frac{1}{n}\sum_{i \in N} E_i = \frac{1}{n}\sum_{i \in N} \frac{\sum_{j \in N, j \neq i} d_{ij}^{-1}}{n-1}$$

To calculate local efficiency $E_{loc,i}$ of node *i* (Latora & Marchiori, 2001; Rubinov & Sporns, 2010), we let $d_{jh}(N_i)$ be the shortest path length between *j* and *h* using only neighbours of *i*, and a_{ij} be the connection (1 as present, 0 as absent) between nodes *i* and *j*:

$$E_{loc} = \frac{1}{n}\sum_{i \in N} E_{loc,i} = \frac{1}{n}\sum_{i \in N} \frac{\sum_{j,h \in N, j \neq i} a_{ij} a_{ih} [d_{jh}(N_i)]^{-1}}{k_i(k_i - 1)}$$

References

Arnatkevičiūtė, A., Fulcher, B. D., & Fornito, A. (2019). A practical guide to linking brain-wide gene expression and neuroimaging data. *NeuroImage*, *189*, 353–367. https://doi.org/10.1016/j.neuroimage.2019.01.011

Fulcher, B. D., & Fornito, A. (2016). A transcriptional signature of hub connectivity in the mouse connectome. *Proceedings of the National Academy of Sciences of the United States of America*, *113*(5), 1435–1440. PubMed. https://doi.org/10.1073/pnas.1513302113

Gorgolewski, K. J., Fox, A. S., Chang, L., Schäfer, A., Arélin, K., Burmann, I., Sacher, J., & Margulies, D. S. (2014). Tight fitting genes: Finding relations between statistical maps and gene expression patterns. *F1000Research*, *5*.

Guimerà, R., & Nunes Amaral, L. A. (2005). Functional cartography of complex metabolic networks. *Nature*, *433*(7028), 895–900. https://doi.org/10.1038/nature03288

Kolberg, L., Raudvere, U., Kuzmin, I., Vilo, J., & Peterson, H. (2020). gprofiler2—An R package for gene list functional enrichment analysis and namespace conversion toolset g:Profiler. *F1000Research*, *9(ELIXIR)*(709). https://doi.org/10.12688/f1000research.24956.2

Latora, V., & Marchiori, M. (2001). Efficient behavior of small-world networks. *Physical Review Letters*, *87*(19), 198701.

Newman, M. E. (2008). The mathematics of networks. *The New Palgrave Encyclopedia of Economics*, *2*(2008), 1–12.

Newman, M. E. J. (2005). A measure of betweenness centrality based on random walks. *Social Networks*, *27*(1), 39–54. https://doi.org/10.1016/j.socnet.2004.11.009

Newman, M. E. J. (2006). Finding community structure in networks using the eigenvectors of matrices. *Physical Review E*, *74*(3), 036104. https://doi.org/10.1103/PhysRevE.74.036104

Newman, M. E. J., & Girvan, M. (2004). Finding and evaluating community structure in networks. *Physical Review E*, *69*(2), 026113. https://doi.org/10.1103/PhysRevE.69.026113

Raudvere, U., Kolberg, L., Kuzmin, I., Arak, T., Adler, P., Peterson, H., & Vilo, J. (2019). g:Profiler: A web server for functional enrichment analysis and conversions of gene lists (2019 update). *Nucleic Acids Research*, *47*(W1), W191–W198. https://doi.org/10.1093/nar/gkz369

Reimand, J., Isserlin, R., Voisin, V., Kucera, M., Tannus-Lopes, C., Rostamianfar, A., Wadi, L., Meyer, M., Wong, J., Xu, C., Merico, D., & Bader, G. D. (2019). Pathway enrichment analysis and visualization of omics data using g:Profiler, GSEA, Cytoscape and EnrichmentMap. *Nature Protocols*, *14*(2), 482–517. https://doi.org/10.1038/s41596-018-0103-9

Rubinov, M., & Sporns, O. (2010). Complex network measures of brain connectivity: Uses and interpretations. *NeuroImage*, *52*(3), 1059–1069. https://doi.org/10.1016/j.neuroimage.2009.10.003

Watts, D. J., & Strogatz, S. H. (1998). Collective dynamics of 'small-world' networks. *Nature*, *393*(6684), 440–442. https://doi.org/10.1038/30918
